# Positive Interactions Under Ocean Warming and Acidification: Crustose Coralline Algae Holobionts Enhance Gorgonian Larval Settlement Under Climate Change

**DOI:** 10.1111/1462-2920.70217

**Published:** 2025-12-14

**Authors:** E. Manea, P. E. Galand, S. Comeau, C. Ferrier‐Pagès, B. Giordano, L. Pezzolesi, J.‐B. Raina, S. N. Elahee Doomun, R. Tignat‐Perrier, L. Bramanti

**Affiliations:** ^1^ Laboratoire d’Ecogéochimie des Environnements Benthiques, LECOB Observatoire Océanologique de Banyuls‐sur‐Mer Centre National de la Recherche Scientifique (CNRS)‐Sorbonne Université Banyuls‐sur‐Mer France; ^2^ Sorbonne Université, CNRS‐INSU, Laboratoire Ddoceanographie de Villefranche France; ^3^ Centre Scientifique de Monaco, Coral Ecophysiology Team and Unité CSM‐Chanel Sur les Coraux Précieux Monaco; ^4^ Department of Life and Environmental Sciences University of Cagliari Cagliari Italy; ^5^ Department of Biological, Geological and Environmental Sciences (BiGeA) University of Bologna Ravenna Italy; ^6^ Interdepartmental Centre for Industrial Research in Renewable Resources, Environment, Sea and Energy (CIRI‐FRAME), University of Bologna Ravenna Italy; ^7^ PSL Université Paris: EPHE‐UPVD‐CNRS, USR 3278 CRIOBE, Université de Perpignan France; ^8^ Metabolomics Australia, Bio21 Molecular Science and Biotechnology Institute, University of Melbourne Parkville Victoria Australia

**Keywords:** CCA, chemical cues, *Eunicella singularis*, microbiome, seawater warming and acidification, species interactions, Western Mediterranean Sea

## Abstract

Crustose coralline algae (CCA) and their bacterial communities can emit chemical cues favoring coral larval settlement. Indeed, larvae of *Eunicella singularis* (white gorgonian) preferentially settle on CCA. Here, we investigated the effect of two Mediterranean CCA holobionts, *Macroblastum dendrospermum* and *Lithophyllum stictiforme*, on *E. singularis* larvae settlement and their bacterial communities, after warming and acidification treatments. We exposed CCA to temperature and pH expected for 2100 (SSP5‐8.5) and to a marine heatwave event. Larval settlement increased 1.8–2.7 times in the presence of CCA exposed to warming and acidification compared to non‐exposed CCA. High abundance of bacteria belonging to the Pirellulaceae family was observed in all CCA, while a higher abundance of monosaccharides was found in exudates of exposed CCA. Based on CCA‐related 16S rDNA metabarcoding and metabolomics results, we hypothesize that the enhanced larval settlement was driven by the Pirellulaceae breakdown and utilization of CCA polysaccharides, in combination with polysaccharide release through the CCA cell walls likely augmented by decalcification. Furthermore, CCA acted as sources of bacterial taxa that may establish and persist in the adult *E. singularis* holobiont, independently of climate change effects. We conclude that CCA are key for *E. singularis* recruitment success, especially under future climate conditions, and contribute to their microbiome development.

## Graphical abstract

Crustose coralline algae (CCA) are key for the settlement of the larvae of the Mediterranean white gorgonian, *Eunicella singularis*, especially after treatment under future climate change conditions. CCA also influence larval microbiome development. (A) Enhanced larval settlement is driven by bacterial breakdown and utilisation of CCA polysaccharides, in combination with polysaccharides released through the CCA cell walls. (B) CCA act as sources of bacterial taxa that can establish and persist in adult *E. singularis* holobionts, independently of climate change effects.

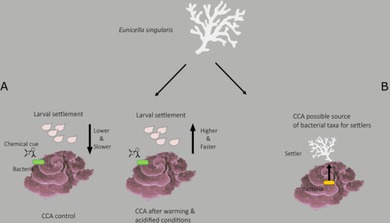

## 1 Introduction

Climate change is significantly altering marine ecosystems by modifying the physico‐chemical properties of seawater, reducing biodiversity, disrupting food web dynamics, shifting species distribution and causing local extinctions. These changes ultimately impact the structure and ecological functioning of marine communities (Hoegh‐Guldberg and Bruno 2010; Pecl et al. 2017; Garrabou et al. 2022). In the Mediterranean Sea, mass mortality events of gorgonians have been repeatedly observed at shallow depths since 1999 (Bramanti et al. 2023). Gorgonian corals are ecosystem engineers that can form three‐dimensional complex habitats known as marine animal forests (Rossi et al. 2017). The canopies of the gorgonian forests provide structural complexity, food and shelter to numerous species, constituting biodiversity hotspots (Nelson and Bramanti 2020; Lasker et al. 2020). In the last decades, several populations of the white gorgonian *Eunicella singularis* have been hit by marine heatwaves (Turicchia et al. 2018; Garrabou et al. 2022), and are now the focus of conservation and restoration initiatives.

Current restoration approaches rely primarily on transplantation of adult fragments (Casoli et al. 2022; Edery et al. 2025), an asexual propagation technique. However, these approaches are constrained by the need for donor populations and the high mortality of transplanted colonies, which result in limited scalability and low genetic diversity of the restored populations (Omori 2019; Moriarty et al. 2020). These limitations could be addressed by techniques based on sexually derived propagules and enhanced larval settlement (Pollock et al. 2017; Ladd et al. 2018; Manea et al. 2023). These sexual propagation techniques, despite the difficulty of getting high numbers of competent larvae to settle (i.e., settlement bottleneck), offer the major advantages of not depending on donor populations, maintaining genetic diversity and being up‐scalable (Chamberland et al. 2017; van Oppen et al. 2017). Despite their benefits, sexual propagation techniques are not applied to restore *E. singularis* , and the knowledge necessary for their development is scant. Developing sexual propagation‐based approaches for the restoration of *E. singularis* is recommended and requires a better understanding of the dynamics driving its early life stages (i.e., larval settlement and metamorphosis), as well as the factors promoting larval settlement and increasing post‐settlement survival.

*E. singularis* is a gonochoric species that undergoes sexual reproduction. Similarly to other brooders, oocytes are fertilized internally and mature lecithotrophic larvae (planulae) are released once a year in summer (end of June/beginning of July). After a pelagic larval duration (PLD) that can last up to 78 days, larvae settle and metamorphose into a primary polyp (Weinberg and Weinberg 1979; Zelli et al. 2020; Guizien et al. 2020). *E. singularis* larval settlement is higher in the presence of crustose coralline algae (CCA) compared to bare rock (Zelli et al. 2020), but the mechanisms behind this substrate preference have not yet been described. Similar larval preferences have been observed in tropical environments, where CCA or their associated bacteria promote settlement and metamorphosis of coral larvae through the production of metabolites that act as chemical cues (Gómez‐Lemos et al. 2018; Siboni et al. 2020; Jorissen et al. 2021; Quinlan et al. 2023). For example, tetrabromopyrrole (TBP) and the red pigment cycloprodigiosin (CYPRO), both produced by specific CCA‐associated *Pseudoalteromonas* (Tebben et al. 2011; Sneed et al. 2014; Fiegel et al. 2025), can influence coral settlement and metamorphosis.

While the mechanisms underlying beneficial interactions between tropical reef‐building corals and CCA holobionts (i.e., CCA and their associated microbial communities) during larval settlement have been at least partially explored, these interactions remain largely unknown for Mediterranean gorgonians. Furthermore, as the negative impacts of climate change are expected to increase through this century, it is important to understand how these environmental changes may affect the CCA–gorgonians interactions.

Past studies on tropical reefs showed that warming and acidification can lead to changes in CCA bacterial assemblages with potential consequences on coral larval settlement, ultimately impacting coral reproductive success (Webster et al. 2011, 2013). Mediterranean CCA are already suffering from the effects of marine heatwaves and pathogens (Hereu and Kersting 2016; Quéré et al. 2019). They are also known to be sensitive to ocean acidification and warming (Martin and Gattuso 2009; Cornwall et al. 2022; Vitelletti et al. 2023), which will intensify in the coming years (Reale et al. 2022). Thus, the potential disruption of the interactions between gorgonian larvae and CCA holobionts deserves consideration. Viladrich et al. (2022) showed that the survival of *E. singularis* larvae and their settlement rate on CCA were unaffected by a simulated heatwave event projected for 2050. However, their study did not investigate the combined effects of ocean acidification and warming on CCA holobionts nor the consequences on larvae–CCA interactions.

Beyond their possible role in facilitating coral larval settlement, bacteria are also key players in the evolution and development of their host (McFall‐Ngai et al. 2013). Coral larvae and settlers can develop their microbiome by acquiring bacteria both vertically (i.e., from parents to offspring) and horizontally (i.e., from the environment) (Lema et al. 2014; Nitschke et al. 2016; Ali et al. 2019; Tignat‐Perrier et al. 2025). The transmission of bacteria involved in the development and survival of coral recruits from CCA holobionts might be an additional interaction promoting the health of corals. Recently, it has been shown that the microbial community of tropical coral settlers changes according to the substrate and macroalgae on which they grow (Hochart et al. 2024). In the Mediterranean Sea, the potential influence of CCA on the bacterial communities of *E. singularis* larvae and settlers remains to be tested.

The general aim of this study is to explore the mechanisms that promote *E. singularis* larval settlement and to test whether warming and acidification impact such mechanisms. Specifically, we measured larval settlement on CCA pre‐exposed to climate change scenarios, characterised CCA‐associated microbial communities and metabolites and assessed the potential transfer of bacteria from CCA to *E. singularis* larvae and settlers. The results of this study shed light on the settlement dynamics of *E. singularis* under different climate change scenarios and inform restoration strategies for this species.

## 2 Material and Methods

### 2.1 Collection of CCA Samples

**FIGURE 1:**
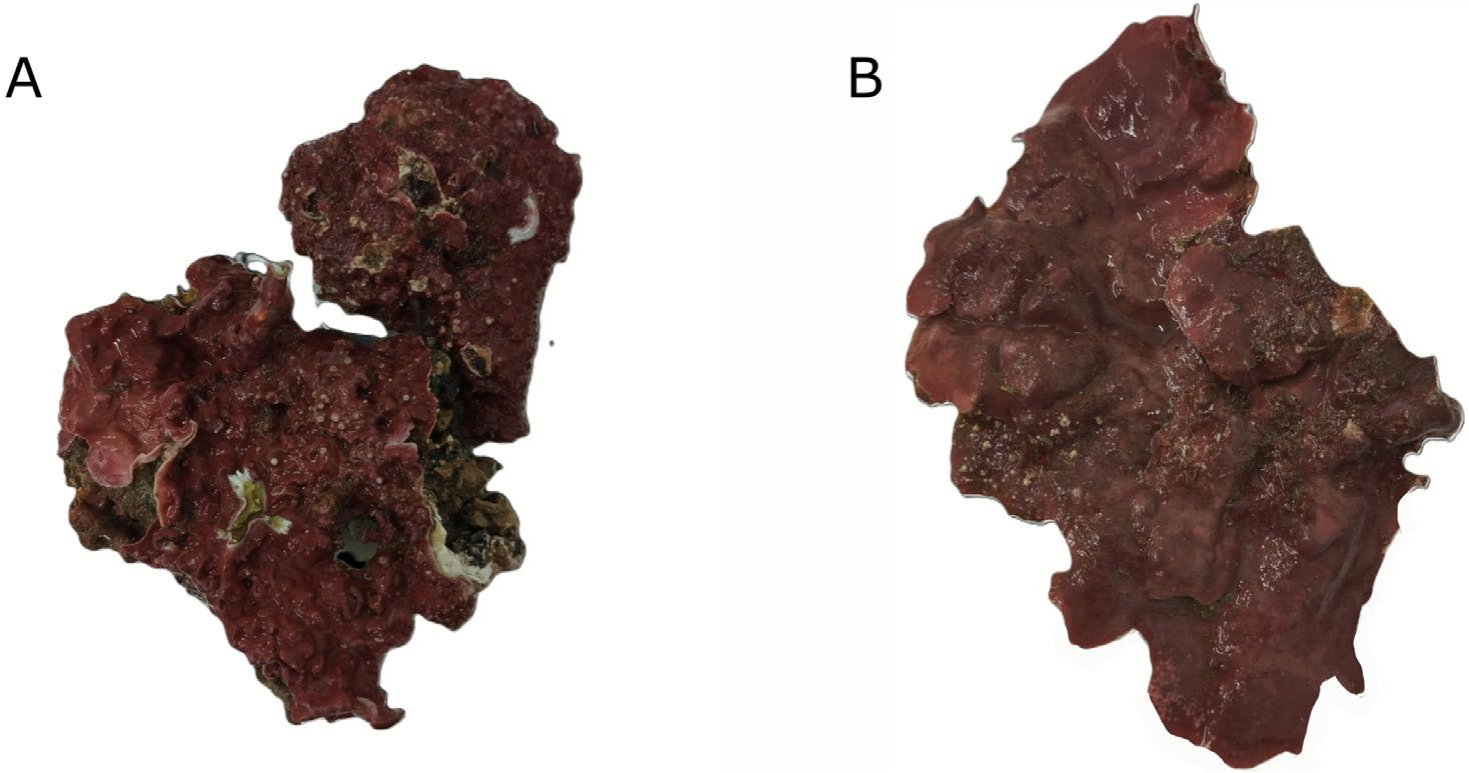
Thalli of the crustose coralline algae (A) *Macroblastum dendrospermum* and (B) *Lithophyllum stictiforme*.

Thalli of the CCA *Macroblastum dendrospermum* and *Lithophyllum stictiforme* (ca. 5–7 cm wide and 5–10 cm long, Figure 1A,B) were collected at the beginning of April 2023 at the Canadells site in Banyuls‐sur‐Mer, France, Western Mediterranean Sea (42°26.900′N, 3°10.347′E), an area that harbours populations of the white gorgonian *E. singularis* . A total of 12 thalli were collected for each CCA species by SCUBA diving using hammer and chisel at 25 m depth within an area of approximately 30 m^2^.

Epiphytes were carefully removed from the CCA surface using sterile forceps. The two CCA were previously identified in the same site combining morphological and molecular techniques (Manea et al. 2025), but given the high degree of cryptic species in CCA, one fragment (ca. 2 cm^2^) of the CCA thalli was air‐dried and stored in plastic bags with silica gel for subsequent DNA extraction and species identification (see Section 2.4 for details). The remaining part of the thalli was kept in open‐circuit aquaria for a week for acclimatisation to the laboratory conditions (see Supporting Information for further details). Only the seven largest thalli for each CCA species (ca. 15 × 8 cm) were sampled for phylogenetic studies.

### 2.2 Experimental Setup and Treatments

Following acclimatisation, a portion (12.6 ± 2.5 cm^2^) of nine of the twelve CCA thalli collected for each species (*M. dendrospermum* and *L. stictiforme*) was placed individually into 1‐L cylindrical open‐circuit aquaria (see Supporting Information for details on CCA acclimatisation and fragment preparation). Each set of three aquaria per CCA species was maintained under one of the three experimental treatments for 56 days prior to larvae introduction:

- Control (CTRL): Ambient conditions (pH_T_ = 8.1, T = 16.1°C).
- T1: Conditions projected for 2100 under the SSP5‐8.5 scenario (pH_T_ = 7.70, T = +2.5°C; Kwiatkowski et al. 2020; Reale et al. 2022).
- T2: Identical to T1 for 35 days, followed by a 21‐day marine heatwave (maximum T = 26°C) based on projections for 2050 (Galli et al. 2017). The heatwave was reproduced by increasing the temperature by 0.5°C per day over 16 days.

**FIGURE 2:**
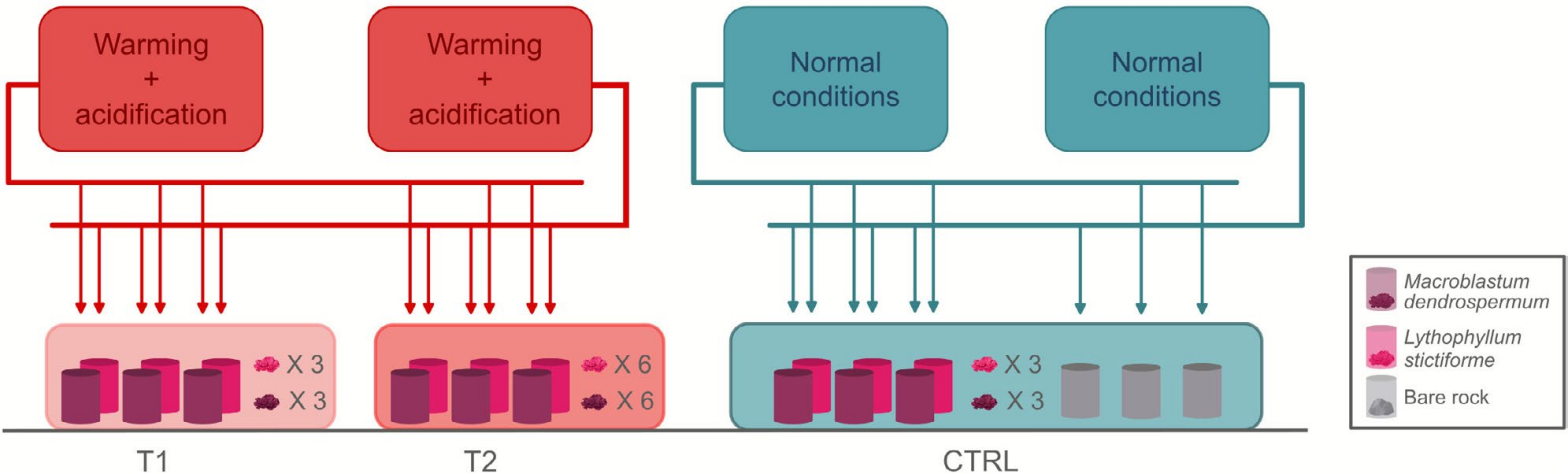
Schematic of the experimental design. T1 = Low pH and warming conditions corresponding to pH_T_ 7.70 and temperature increase of 2.5°C for 56 days. T2 = Low pH and warming conditions corresponding to pH_T_ 7.70 and temperature increase of 2.5°C for 35 days followed by a 21‐day heatwave phenomenon at 26°C. CTRL = normal conditions of water pH and temperature. For each treatment, six cylinders (three replicates per algal species), containing a portion of 12.6 ± 2.5 cm^2^ of CCA, were placed inside three larger aquaria (‘water baths’), each dedicated to one of the experimental treatments. Three pieces of combusted bare rock of the same dimension were placed in three cylinders within the CTRL. The remaining parts of each CCA thalli present in the cylinders were placed in the respective water baths for bacterial community analysis at the end of the experimental treatments. Three intact thalli per algal species were kept in the T2 water baths for exo‐metabolite analysis after 56 days of treatment exposure. Four 100‐L tanks, header tanks, two at low pH and warming conditions and two at normal conditions of water pH and temperature, randomly supplied the cylinders dedicated to each treatment, respectively. The total time of CCA exposure to the different treatments was 56 days, followed by 59 days of larval settlement.

The experimental setup consisted of three large aquaria (‘water baths’), each dedicated to one of the experimental treatments (T1, T2, CTRL; Figure 2) and each contained six cylinders (three replicates per CCA species). To assess bacterial community composition at the end of the treatment period, the remaining part of the nine CCA thalli was maintained in the three respective water baths (i.e., the same three replicates per algal species). Additionally, three pieces of bare rock were placed in three cylinders within the CTRL water bath after being combusted at 459°C for 4 h (to remove organisms and organic matter) and prepared similarly to the CCA fragments (see Supporting Information). The 21 cylinders (three per CCA species and per treatment, plus three with bare rock) received a continuous water flow of 50 mL min^−1^, delivered by gravity from four 100‐L tanks (‘header tanks’) that randomly supplied them. The header tanks were supplied with seawater pumped from a depth of 14 m in Banyuls‐sur‐Mer Bay and filtered through a 5‐μm filter before distribution. The remaining three thalli per CCA species out of the 12 were kept intact in the T2 water bath and sampled at the end of the treatment for exo‐metabolite analysis (Figure 2).

Throughout the 56‐day treatment period, pH and temperature were regulated using dedicated controllers and probes (APEX Neptune Systems, www.neptunesystems.com). Temperature, pH, salinity and total alkalinity were measured regularly (see Supporting Information for details) and used to calculate the other carbonate chemistry parameters using the R package ‘*seacarb*’ with the *carb* function (Gattuso et al. 2024). LED (Sera LED cool daylight 1420 mm/27 W) was used to simulate seasonal variations in daily irradiance at the sampling site (see Supporting Information for further details).

After 56 days of exposure to the treatments, temperature and pH were returned to ambient conditions, and half of the CCA thalli kept in the water baths were sampled and frozen immediately at −80°C for subsequent analysis of the associated bacterial communities.

### 2.3 Gorgonian Larval Collection and Settlement Assay

Gorgonian larvae were obtained as described in Zelli et al. (2020). Briefly, mature colonies of *Eunicella singularis* were collected in late June in Banyuls‐sur‐Mer and maintained in open circuit aquaria at in situ temperature (T = 16.1°C). Once the sex of each colony was determined, the specimens were rearranged in the aquaria to ensure oocyte fertilisation. The aquaria were inspected daily for larval release, which started on the 19th and ended on the 30th of June 2023. After larval release and collection, *E. singularis* colonies were successfully transplanted in the Banyuls‐sur‐Mer public aquarium.

A total of 5460 larvae were collected, of which 260 were randomly added to each CCA experimental cylinder (Figure 2), after 6 to 16 days after collection, which correspond to the pre‐competence period (7–14 days; Padrón et al. 2018). The settlement experiment was conducted on CCA that were previously kept under experimental treatment for 56 days (see above). During the first 20 days, the number of settlers was recorded daily, then every 2 to 3 days until the end of the experiment. In line with Zelli et al. (2020), we defined (i) ‘settlement event’ when the larva stopped their swimming activity to cling onto the substrate, and (ii) ‘settler’ when the larva underwent physiological and morphological differentiation into a polyp (i.e., metamorphosis). The settlement experiment ended after 59 days, when almost all larvae settled or died, and only a few of them (~5) remained in each cylinder.

At the end of the experiment, three to four larvae and settlers were collected for each treatment condition and CCA species. To avoid contamination with the microbiome associated with the substrates, settlers were collected by cutting their base with sterile tweezers and scalpels (see samples' metadata in Table S1). For larvae collection, we avoided the intake of surrounding seawater to prevent contamination. The CCA fragments of the respective aquaria and treatment were also collected and immediately frozen at −80°C, while larvae and settlers were directly put into 50 μL of the extraction buffer of the direct qPCR ProbesMaster kit (Jena Bioscience) and frozen for subsequent analysis of the associated microbiome.

### 2.4 CCA DNA Extraction, Amplification, Sequencing and Phylogenetic Analyses

Briefly, CCA DNA extraction was performed as in Manea et al. (2025) using the Qiagen DNeasy Blood & Tissue Kit (Qiagen, Crawley, UK) with modifications by Broom et al. (2008). The *psb*A gene was PCR‐amplified in both CCA species according to Pezzolesi et al. (2017, 2019), while the mitochondrial COI‐5P fragment was PCR‐amplified in *M*. *dendrospermum* following Peña et al. (2015). DNA sequencing was performed by BMR Genomics (Padua, Italy). The quality of the sequences was assessed by visual inspection of the electropherograms using the Chromas software (Version 2.6.6; Technelysium Pty LTD, South Brisbane, Australia). The alignment was performed using ClustalW and default settings, and phylogeny was constructed using the MEGA software (Version 11.0.13, www.megasoftware.net; Tamura et al. 2021). Neighbor‐Joining (NJ) distance analyses were performed on all data sets using the Maximum Composite Likelihood model in MEGA software, with nodal support assessed by 1000 bootstrap (BR) resamplings. Phylogenetic relationships were inferred using Maximum likelihood (ML) analyses in MEGA software, under a generalised time‐reversible with gamma+invariant sites heterogeneity model (GTR + G + I), and a generalised time‐reversible gamma distributed (GTR + G) alignments, for the COI‐5P and *psbA* alignments, respectively. The bootstrap consisted of 1000 replicates with the complete deletion option (i.e., eliminating the positions containing gaps and missing data) (Saitou and Nei 1987; Nei and Kumar 2000; Pezzolesi et al. 2019; Manea et al. 2025). For more details, see Supporting Information.

### 2.5 Bacteria DNA Extraction, Sequencing and Sequence Analysis

Bacteria DNA from frozen CCA samples and control bare rock fragments was extracted as described in Manea et al. (2025) using a Power Soil DNA Isolation kit according to the manufacturer's instructions (QIAGEN, Hilden, Germany). Before sequencing, the quality of DNA was controlled by quantification with a DeNovix DS11 Spectrophotometer—Fluorometer (DeNovix Inc., Delaware). Analyses of the prokaryotic 16S rDNA sequences were conducted by amplifying the bacterial V3‐V4 region, with the forward primer 341F 5′‐CCTACGGGNGGCWGCAG‐3′ and the reverse primer 785R 5′‐GACTACHVGGGTATCTAATCC‐3′ (Klindworth et al. 2013), and sequencing the PCR product on the Illumina MiSeq V3 platform at the Integrated Microbiome Resource (IMR, imr.bio, Dalhousie University, Halifax, Nova Scotia, Canada) to obtain 2 × 300 paired‐end sequences.

For larvae and settlers, a direct PCR approach was chosen to avoid losing the small quantity of bacterial DNA present in single larva and settler samples (see Tignat‐Perrier et al. 2025 and details in Supporting Information). Briefly, after collection, samples were directly put in extraction buffer of the direct qPCR ProbesMaster kit (Jena Bioscience) to lyse the cells. Cell lysis was aided by 20 min of Proteinase K digestion (0.1 μL; 600 U/mL; Qiagen) at 65°C before amplification of the 16S rRNA gene. The V3‐V4 region of the 16S rRNA gene was amplified in three replicates per sample by using the direct qPCR ProbesMaster kit. The three PCR reactions per sample were pooled and amplicons were purified using AMPure XP beads (Beckman Coulter). Negative controls (i.e., PCR without sample material) were processed at the same time as the samples. Amplification was checked using the Bioanalyzer DNA 1000 kit (Agilent), and samples showing amplification were sent to STABvida (Portugal) for library preparation and sequencing following Illumina's standard ‘16S Metagenomic Sequencing Library Preparation’ protocol (Illumina 2013).

Bacterial sequences were processed with the DADA2 package, version 1.16 (Callahan et al. 2016) in R v.4.2.3 (R Core Team 2023). After inspection of quality control profiles, the filtering parameters were set as follows: truncLen = c(260,220), trimLeft = c(17,21), maxN = 1, maxEE = c(3,3), truncQ = 2, rm.phix = TRUE, compress = TRUE. Sequences were denoised with the *dada* function, reads were merged with the *mergePairs* function, and chimeras were removed with *removeBimeraDenovo*. Representative amplicon sequence variants (ASVs) were classified against the SILVA version 138.1 database (McLaren and Callahan 2021). Chloroplast, mitochondria, unknown ASVs at the phylum level and singletons were removed with the R package ‘*phyloseq*’ (McMurdie and Holmes 2013). A total of 6,213,093 reads and 13,908 ASVs were retained (Table S1). No sequences were obtained from rocky samples.

### 2.6 CCA Metabolite Sampling, Extraction and Composition

Samples for CCA's exo‐metabolite characterisation were collected in the water bath dedicated to T2 treatment after 56 days of treatment exposure (i.e., warming and acidification followed by heatwave). Three samples for each CCA species were also collected in situ at the same sampling site (Canadells site) and used as controls for comparison. In both cases, 200 μL of seawater was collected using sterile syringes on the surface of each CCA thallus, for a total of 12 samples (six in situ samples, hereafter referred to as ‘in situ’, and six samples in T2 treatment aquarium). Exo‐metabolites were extracted using solid‐phase extraction (SPE) following a protocol adapted from Dittmar et al. (2008) and analysed using a 2030 Shimadzu gas chromatograph and a TQ8050 quadrupole mass spectrometer (Shimadzu). Both chromatograms and multiple reaction monitoring (MRM) productions were evaluated using the Shimadzu GCMS LabSolutions Insight software (version 3.6). Resulting area responses were normalised to the area response of the internal standard ^13^C_6_‐sorbitol. The full protocol is described in Supporting Information.

### 2.7 Data Analysis and Statistics

#### 2.7.1 Effect of Substrate and Treatment on Larval Settlement and Settlement Timing

The effects of substrate type and experimental treatments on *E. singularis* larval settlement were assessed with two separate statistical analyses due to differences in substrate characteristics and the applicability of the pre‐conditioning treatments (i.e., T1 and T2 treatments not applied on bare rock). First, the effect of substrate type was tested with a one‐way ANOVA with the experimental design including the three substrate types (i.e., bare rock and the two CCA species). Settlement was measured as the total number of settlement events recorded over the 59‐day experimental period. Secondly, the effect of experimental treatments was tested with a two‐way ANOVA conducted with substrates (2 levels: *L. stictiforme* and *M. dendrospermum*) and treatments (3 levels: CTRL, T1 and T2) as independent factors. Bare rock was excluded from this analysis because this substrate was sterilised and not subjected to the experimental treatments.

To determine the effect of substrate and treatment on larval settlement timing, the day of first settlement, recorded for each substrate and treatment in the three replicate aquaria, was used as the response variable. Two separate ANOVA models were applied to analyze the data. The effect of substrate was tested with a one‐way ANOVA based on an experimental design that included the three substrates (i.e., bare rock and the two CCA species). The effect of experimental treatments was tested with a two‐way ANOVA based on an experimental design that included two independent factors: substrate (2 levels: *L. stictiforme* and *M. dendrospermum*) and treatment (3 levels: CTRL, T1 and T2).

For all statistical analyses, ANOVA assumptions (normality and homogeneity of variance) were verified through visual inspection of residuals. When a significant effect was detected, post hoc Tukey's Honest Significant Difference (HSD) comparisons were conducted to identify differences between groups. All statistical analyses were performed using R v.4.4.1 (R Core Team 2024).

#### 2.7.2 Bacterial Communities Associated With CCA and Their Horizontal Transmission to *E. singularis* Larvae and Settlers

A multidimensional scaling ordination (MDS), based on the Bray‐Curtis distance matrix computed from Hellinger transformed data, was constructed with the ‘*vegan*’ package (Oksanen et al. 2007) to visualise similarities in bacterial community composition. After testing the multivariate homogeneity of group dispersions (variances), considering the three CCA treatments (CTRL, T1 and T2) as groups, with the *betadisper* function of the ‘*vegan*’ package, the permutational multivariate analysis of variance (PERMANOVA) was used to test for significant differences in bacterial community composition using 999 permutations under a reduced model with the same package.

Significant differences in community composition at the genus level between each CCA treatment (CTRL, T1 and T2) were tested after checking the normal distribution of the relative abundance values of each genus through a Shapiro–Wilk test with the *shapiro. test* function of the ‘*stats*’ package. When the normality assumption was respected, a one‐way ANOVA with the *aov* function of the same package was applied, followed by a Tukey test for pairwise comparisons. In the case of non‐normal data, a Kruskal‐Wallis test followed by a Dunn test (‘*dunn. test*’ package; Dinno and Dinno 2017) with the Benjamini‐Hochberg method was performed.

To determine the ASVs associated with particular treatment groups, we used the indicator species analysis using the *multipatt* function of ‘*indicspecies*’ package (De Cáceres and Legendre 2009), with func = IndVal using 999 permutations on ASV with ≥ 2% relative abundance. To assess the positive or negative correlation of each ASV in each treatment condition, the Z‐score was calculated with the *scale* function of the ‘*base*’ package. To obtain more information on host association and potential functional traits, an in‐depth taxonomic analysis was carried out of each ASV through a manual BLAST search against the NCBI nucleotide library (https://blast.ncbi.nlm.nih.gov/Blast.cgi).

Significant differences in bacterial community richness between settlers and larvae for each treatment were tested with a one‐way ANOVA and a Tukey test for pairwise comparisons, or a Kruskal‐Wallis test followed by a Dunn test depending on the normality Shapiro–Wilk test results.

To detect potential horizontal transmission of bacteria from CCA to larvae and settlers, the core microbiome (i.e., ASVs shared between the compared individuals; Hernandez‐Agreda et al. 2018) was identified using the ‘*microbiome*’ package (Lahti et al. 2017) and the *core_members* function (Salonen et al. 2012) with detection = 0.01 (with relative abundance ≥ 1%) and prevalence = 0.75 (present in at least 75% of samples). This analysis was applied to CCA, settlers and larvae. In all the analyses that examined the settlers, the settlers attached to the surface of the algae and those on the surface of the aquarium were analysed separately. All statistical analyses were performed in R v.4.4.1 (R Core Team 2024).

#### 2.7.3 Metabolomics

Analyses were performed with MetaboAnalyst 6.0 (Xia et al. 2009; new.metaboanalyst.ca). To assess the differences between the exo‐metabolite profiles associated with T2‐treated CCA (i.e., warming and acidification followed by heatwave) and CCA collected in situ (used as controls), the Significance Analysis of Metabolites (SAM) tests were performed on log‐normalised and mean‐centered data to identify the molecules differently present (raw *p*‐value ≤ 0.05) in the exudates' profiles.

## 3 Results

### 3.1 CCA Taxonomy and Experimental Conditions

The molecular phylogeny analysis based on the *psbA* marker confirmed that all 7 samples collected in this study, which were visually identified as *M. dendrospermum*, clustered with a Mediterranean *M. dendrospermum* collected in Spain, with the Uncultured Corallinales clone LBC0055 collected close to the study area, and with other *M. dendrospermum* collected in the sampling area of this study (Manea et al. 2025) (Fig. S1). Results based on the COI‐5P marker were congruent with the *psbA* tree and grouped the 4 sequences generated within a large clade of Mediterranean *M. dendrospermum* with high support (99%), separated from the other *Mesophyllum* species (Fig. S2). As for *L. stictiforme*, the analysis of the *psbA* marker confirmed that all the 7 samples belonged to the clades 3B of the complex, which included individuals previously collected in this area (Manea et al. 2025), and others from Italy and France (Figure S3).

Regarding the experimental conditions, the CTRL treatment was maintained at a mean pH_T_ of 8.1 ± 0.1 (mean ± SD, *n* = 88), while T1 and T2 (both acidification and warming treatments, with T2 including a heatwave) were maintained at a mean pH_T_ of 7.7 ± 0.1 (mean ± SD, with *n* = 70 and *n* = 21 respectively, Table S2). The CTRL treatment was maintained at 16.1°C ± 1.2°C, the T1 at 18.1°C ± 0.7°C (mean ± SD), and the T2 mean temperature was 23.3°C ± 3°C (average that includes the data of the period of gradual increase in temperature, from 18°C to 26°C). Salinity and total alkalinity were stable for the duration of the experiment, with an average salinity of 37.9 ± 0.2 in all treatments and a total alkalinity ranging between 2560 ± 15 and 2581 ± 33 μmol kg^−1^ in CTRL and T1, respectively (Table S2).

### 3.2 Settlement Dynamics of the White Gorgonian Larvae

**FIGURE 3:**
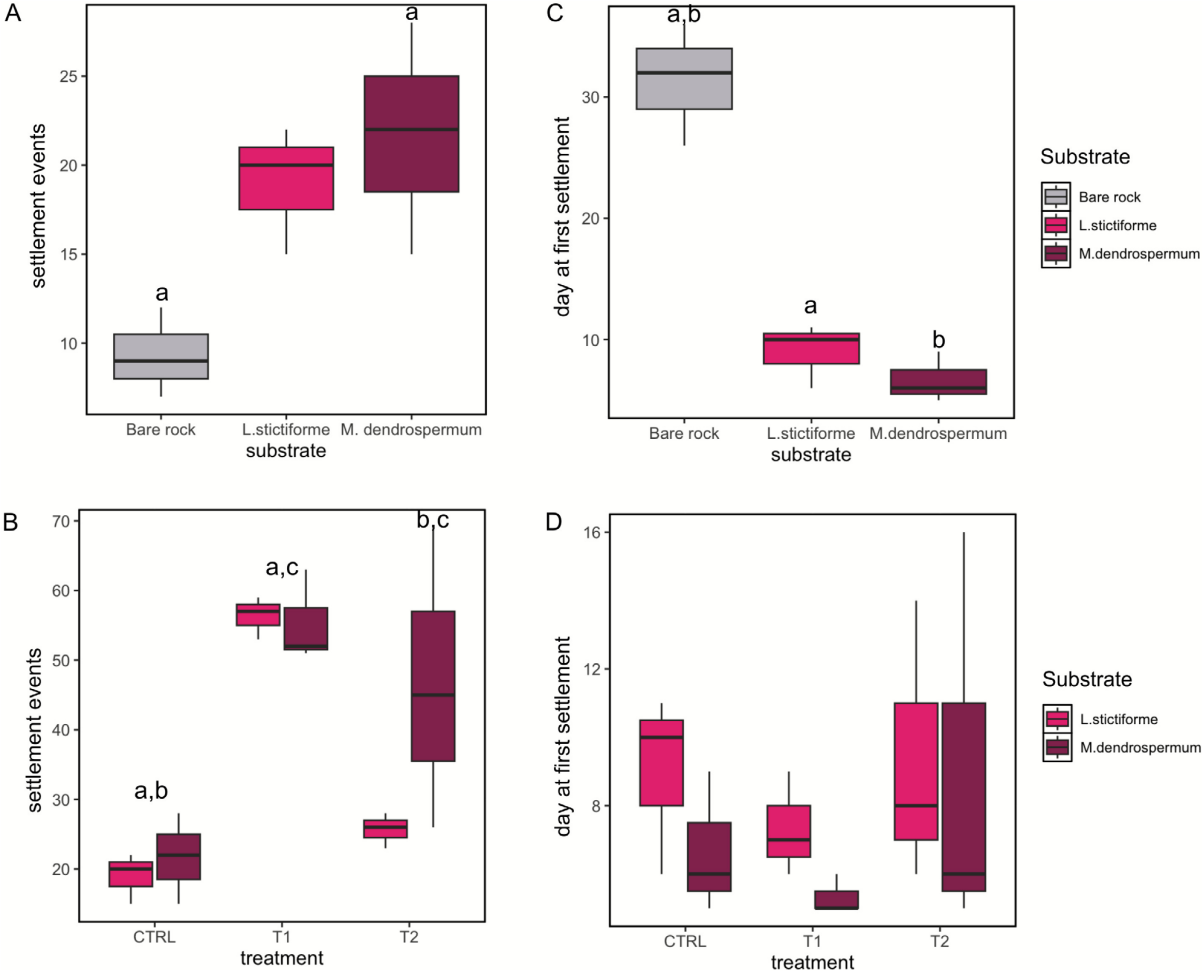
Boxplots reporting the average number of settlement events of the larvae of *Eunicella singularis* at the end of the larval settlement period in the presence of the different substrates (bare rock, and the two CCA species, *Lithophyllum stictiforme* and *Macroblastum dendrospermum*): (A) in control conditions; and (B) with CCA previously subjected to control conditions (CTRL), low pH and warming (T1) and low pH and warming followed by a heatwave event (T2). Boxplots reporting the average day of first settlement event in the presence of (C) the three substrates and (D) the CCA previously subjected to the three treatments. Significant differences between groups analysed by means of post hoc Tukey's Honest Significant Difference (HSD) comparisons are indicated in each graph using letters.

The number of settlement events varied with the substrates (F_(2,6)_ = 6.15; *p* = 0.035). In particular, in CTRL, settlement events were 2.3 times higher on the CCA *M. dendrospermum* than on bare rock (*p* = 0.036, Figure 3A). They doubled on CTRL *L. stictiforme* compared with bare rock, but the difference was not statistically significant (*p* = 0.08; Table S3). No significant differences were found between the number of settlement events on *L. stictiforme* and *M. dendrospermum* (33.6 ± 17.9 and 41.2 ± 19.1, respectively; *p* = 0.76).

The number of settlement events was significantly different among treatments (F_(2,15)_ = 15.53; *p* < 0.001), with 2.7 times more events under T1 conditions (i.e., warming and acidification, 55.8 ± 4.6) and 1.8 times more events under T2 conditions (i.e., warming and acidification followed by heatwave, 36.1 ± 17.9) compared to CTRL (20.3 ± 4.9, *p* < 0.001 for T1 vs. CTRL; *p* < 0.05 for T2 vs. CTRL; Figure 3B, Table S4) for both CCA. In addition, the number of settlement events under T1 conditions was higher than under T2 conditions (*p* = 0.012).

For both CCA species, larvae settled and metamorphosed preferentially on the CCA surface rather than on the aquarium walls. In particular, considering the total number of larvae distributed in each experimental system, 1%, 4% and 13% of larvae settled in CTRL, T2 and T1, respectively. Of these, 54%, 58% and 76% of larvae settled on the CCA surface.

We detected a significant effect of substrate type on the timing of settlement (one‐way ANOVA, F_(2,6)_ = 45.52; *p* < 0.001). Settlement took place more than three weeks earlier on *M. dendrospermum* and *L. stictiforme* than on bare rock (*p* < 0.001, Figure 3C, Table S5). The first settlement event in the control condition occurred 7.8 ± 2.4 days after the start of the experiment, while under T1 and T2 conditions, larvae settled after 6.3 ± 1.5 and 9.1 ± 4.6 days, respectively (Figures 3D and S4). Neither CCA species (F_(2,12)_ = 0.96, *p* = 0.347) nor treatment (F_(2,12)_ = 1.06, *p* = 0.377) had significant effects on the day of first settlement, and no significant interaction was detected between substrate and treatment (F_(2,12)_ = 0.15, *p* = 0.861).

### 3.3 Bacterial Communities of the CCA Under Different Experimental Conditions

**FIGURE 4:**
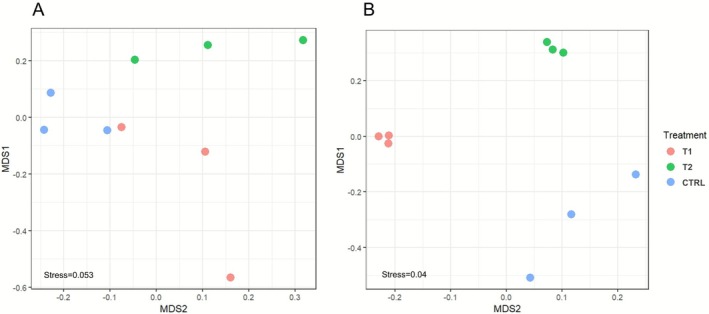
CCA bacterial community composition. Multidimensional scaling ordination (MDS) based on Bray‐Curtis distance of bacterial communities associated with the CCA (A) *Macroblastum dendrospermum* and (B) *Lithophyllum stictiforme* after 56 days under control conditions (CTRL), acidification and warming (T1) and acidification and warming followed by a heatwave event (T2).

The bacterial community composition of the CCA at the ASV level differed significantly between treatments for both algal species (*p* < 0.005, Figure 4A,B, Table S6). There was less within‐treatment dispersion in community composition in *L. stictiforme* than in *M. dendrospermum* (Figure 4).

**FIGURE 5:**
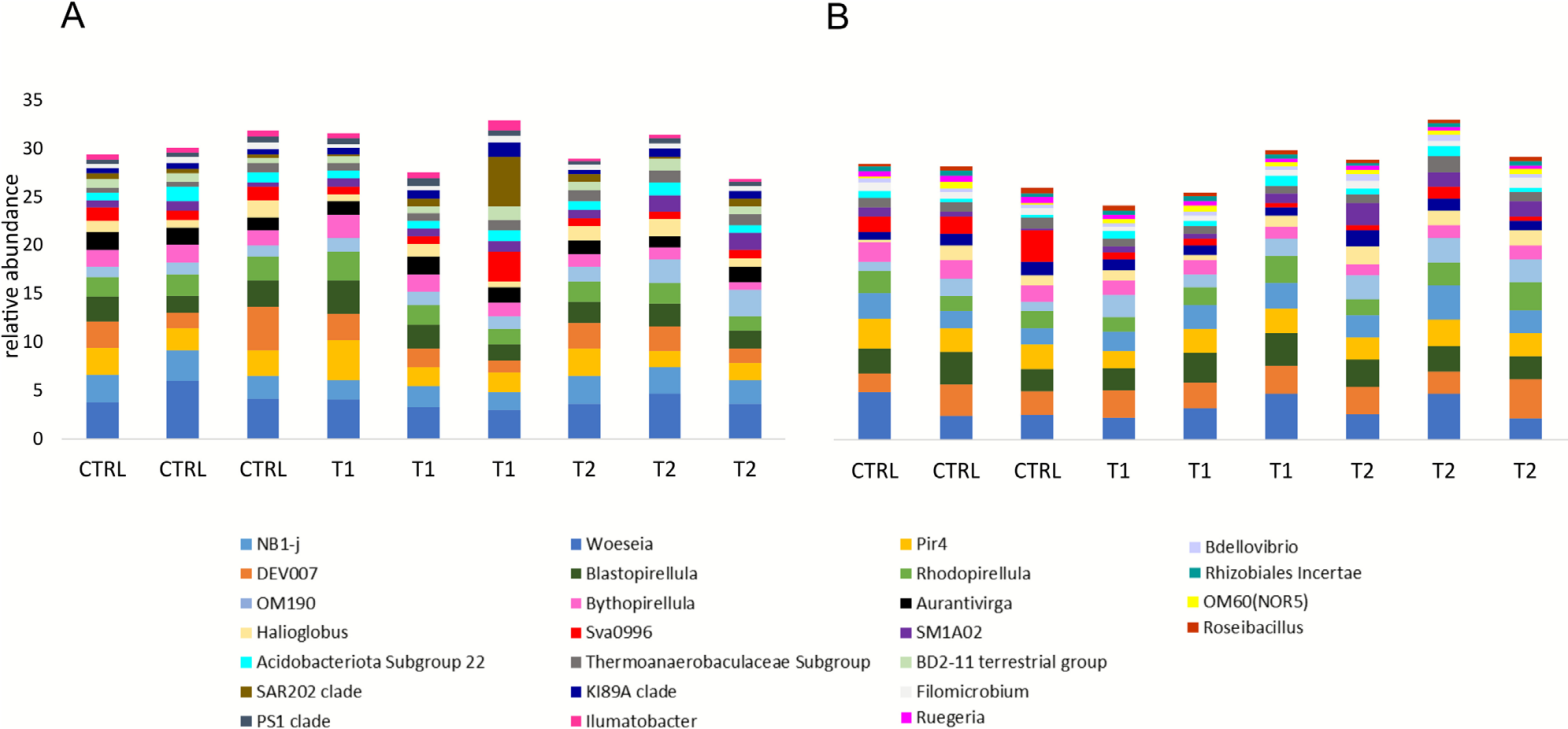
Taxonomic composition of the CCA‐associated bacteria at the genus level. Relative abundance of the 20 most abundant bacterial genera in the CCA (A) *Macrobalstum dendrospermum*, and (B) *Lithophyllum stictiforme* under control conditions (CTRL), acidification and warming (T1), and acidification and warming followed by a heatwave event (T2).

The taxonomic analysis, carried out at the genus level, revealed that both *M. dendrospermum* and *L. stictiforme* samples predominantly hosted the NB1‐j group (average 4% ± 0.6% in *M. dendrospermum*, and 3.3% ± 0.1% in *L. stictiforme*), *Woeseia* belonging to the Gammaproteobacteria (average 2.5% ± 0.4% in *M. dendrospermum*, and 2.4% ± 0.3% in *L. stictiforme*), and the Verrucomicrobium DEV007 group (average 2.3% ± 0.5% in *M. dendrospermum*, and 2.8% ± 0.2% in *L. stictiforme*; Figure 5A,B). Four genera of the family Pirellulaceae (class Planctomycetes), *Blastopirellula*, Pir4 lineage, *Rhodopirellula*, *Bythopirellula* were also abundant in samples from both CCA species. The contribution of these shared taxa did not change between CCA species and treatments. Bacteria belonging to the OM190 group increased in T2 compared to CTRL in both CCA (2.2% ± 0.6% in *M. dendrospermum* and 2.5% ± 0.1% in *L. stictiforme*, *p* < 0.05; Figure 5A,B). The SM1A02 also increased in T2 compared to both T1 and CTRL in *L. stictiforme* (1.7% ± 0.4%, *p* < 0.05; Figure 5B). In *M. dendrospermum*, the *Thermoanaerobaculaceae* subgroup was also significantly more abundant in T2 compared to CTRL, contributing 1.2% ± 0.07%, while the Sva0996 was significantly less abundant (*p* < 0.05; Figure 5A).

**FIGURE 6:**
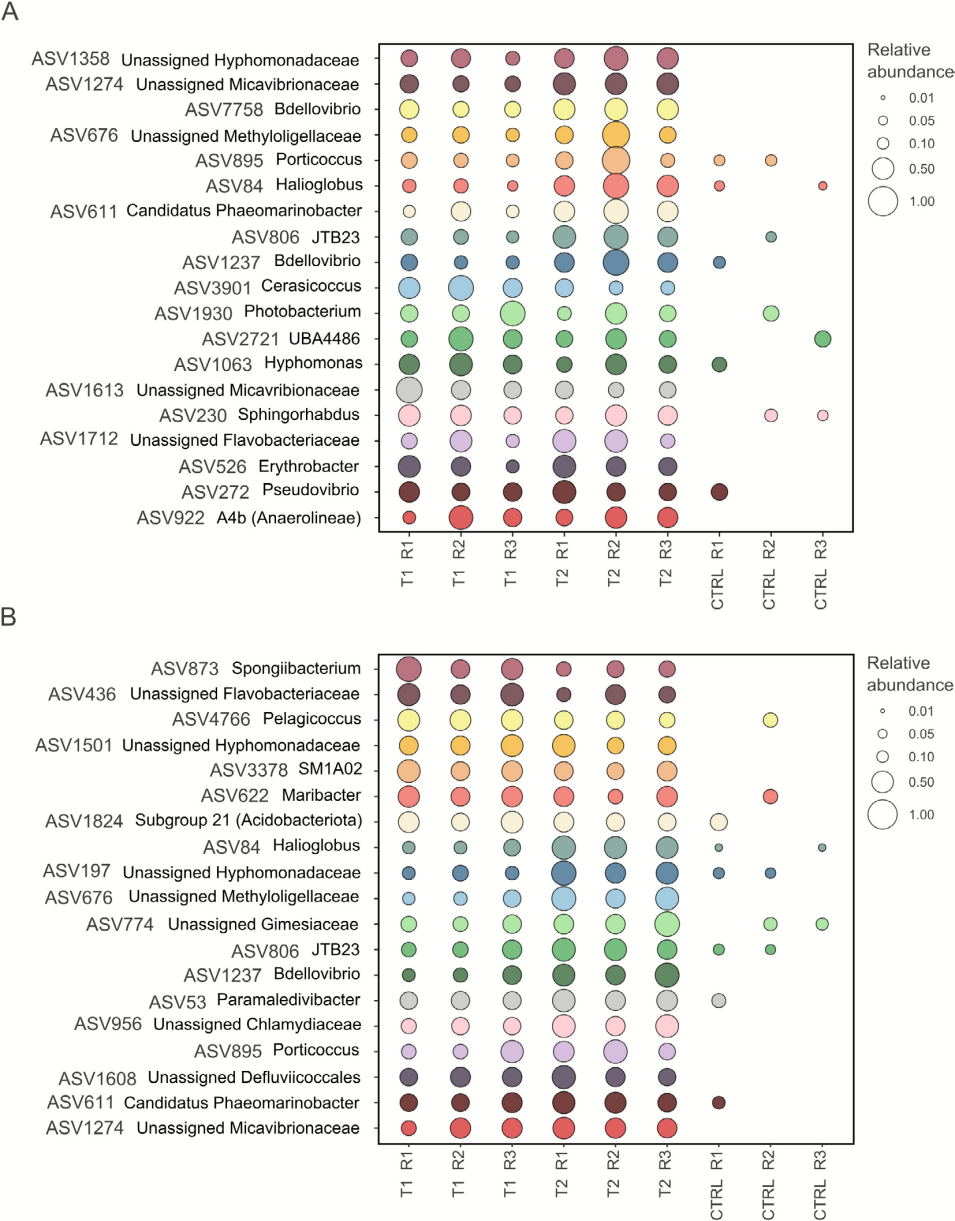
*Macroblastum dendrospermum* and *Lithophyllum stictiforme* ASVs significantly correlated with acidification and warming treatments. (A) Relative abundance of ASVs associated with *M. dendrospermum* samples under control conditions (CTRL), acidification and warming (T1), and acidification and warming followed by a heatwave event (T2). (B) relative abundance of ASVs associated with *L. stictiforme* samples under the three treatments.

The indicator species analysis identified a total of 19 and 55 ASVs significantly associated with warming and acidification treatments in *M. dendrospermum* and *L. stictiforme*, respectively. Among the 19 ASVs with the highest statistical significance for both CCA species, there was a significant increase of ASV806 belonging to the order JTB23 in both CCA species subjected to T2 (*p* < 0.05), and an increase of seven ASVs belonging to the family Pirellulaceae in *L. stictiforme* samples subjected to T1 and T2 conditions (*p* < 0.05; Figure 6A,B; Tables S7 and S8).

### 3.4 CCA Metabolites

**FIGURE 7:**
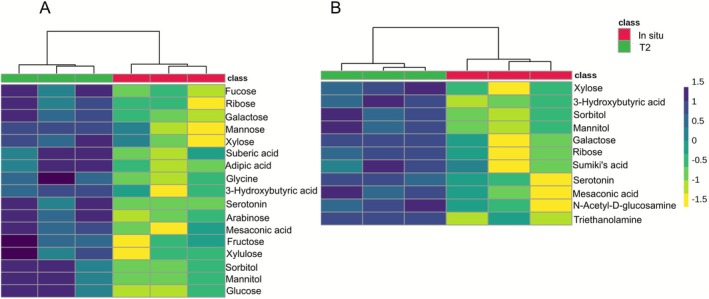
Heatmaps showing the CCA exo‐metabolites significantly different between in situ conditions and the T2 treatment (acidification and warming followed by heatwave) in (A) *Macroblastum dendrospermum* and (B) *Lithophyllum stictiforme*.

A total of 98 compounds were identified in the exudates (exo‐metabolomes) of the two CCA (Table S9). Among these, 17 and 11 compounds were significantly more abundant after T2 treatment (acidification and warming followed by heatwave) in *M. dendrospermum* and *L. stictiforme*, respectively (*p* value < 0.05; Figure S5A,B; Table S10). In both species, there was a significantly higher abundance of monosaccharides, including galactose, ribose and xylose (*p* value < 0.05; Figure 7A,B). The exudates of both CCA also contained significantly more serotonin, mesaconic acid, sorbitol, mannitol and 3‐hydroxybutyric acid (*p* value < 0.05). *M. dendrospermum* exudates under T2 treatment contained a significantly higher abundance of glucose, arabinose, fucose, xylulose, fructose and mannose (*p* value < 0.05; Figure 7A).

### 3.5 Bacterial Communities of White Gorgonian Larvae and Settlers and CCA

**FIGURE 8:**
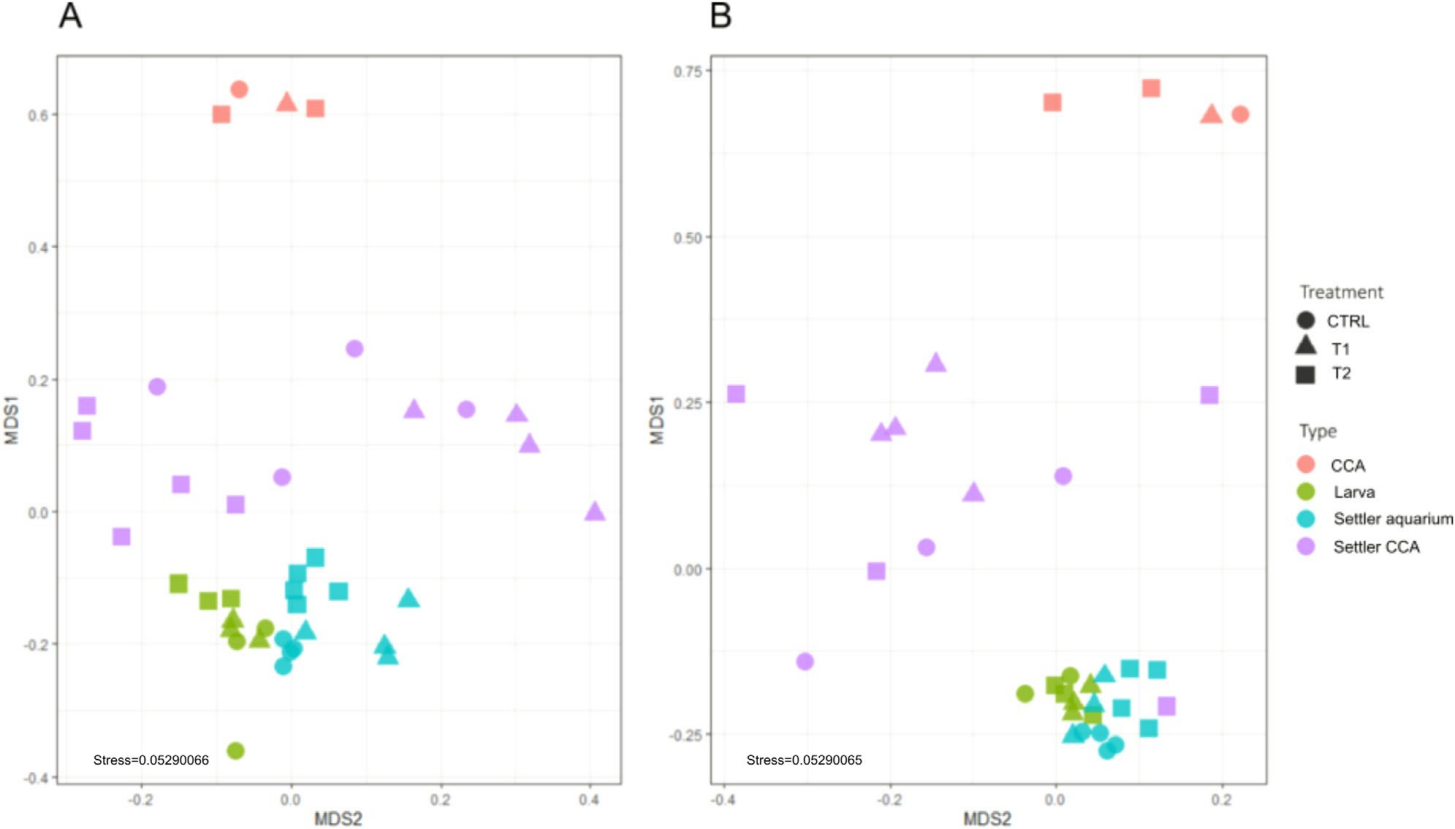
Multidimensional scaling ordination (MDS) based on Bray‐Curtis distance of bacteria communities associated with the CCA, white gorgonian larvae, settlers on the aquarium walls and settlers on CCA under control conditions (CTRL), acidification and warming (T1) and acidification and warming followed by a heatwave event (T2). (A) Aquaria with *Macroblastum dendrospermum* and (B) with *Lithophyllum stictiforme*.

Overall, the bacterial community composition was significantly different between larvae, settlers and CCA for both CCA species (*p* < 0.001or *p* < 0.01, Table S11). More precisely, the MDS showed that bacterial communities clustering was driven by holobiont type rather than treatment. Bacterial communities associated with settlers on the walls of the aquaria and swimming larvae were more similar to each other for both CCA species than bacterial communities of CCA and settlers on algae (Figure 8A,B). Comparison between alpha‐diversity values (i.e., richness) showed significantly higher values associated with settlers on *M. dendrospermum* thalli compared to larvae (*p* < 0.05) and with settlers on *L. stictiforme* thalli compared to both settlers on aquaria surfaces and larvae (*p* < 0.01, Figure S6).

Overall, the 20 most abundant ASVs of the bacterial communities associated with settlers and larvae belonged to Alpha‐ and Gammaproteobacteria (Figure S7). The less common classes were more diversified and did not show a specific distribution pattern between recruit types and CCA conditions (Figure S7). In most of the larvae and settlers, ASVs of the classes Spirochaetia, Alphaproteobacteria and Bacteroidia belonged to the orders Spirochaetales, Rickettsiales and Flavobacteriales, respectively, in line with previous observations on *E. singularis* adults' microbiome (van de Water et al. 2017).

**FIGURE 9:**
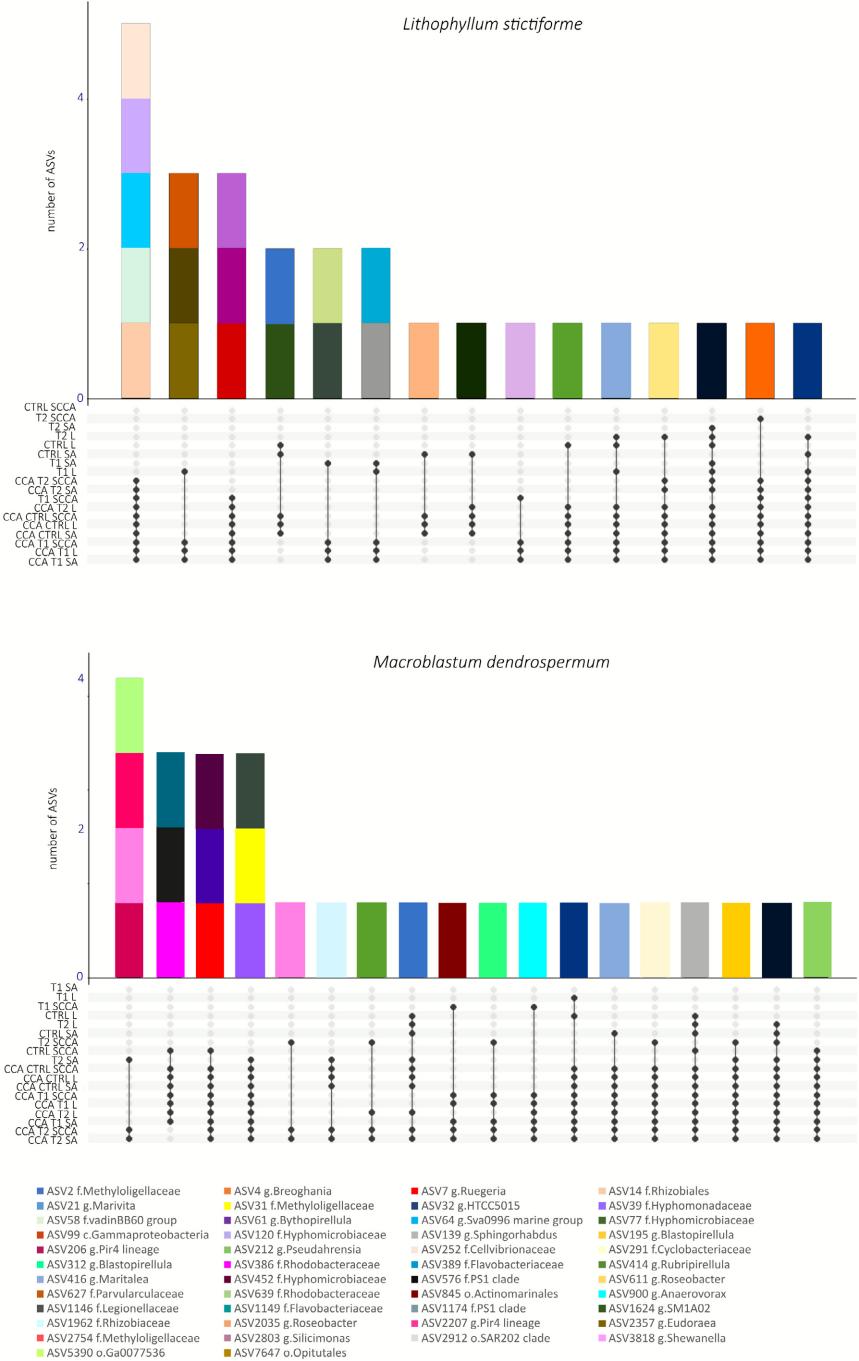
ASVs shared between *Eunicella singularis* settlers on CCA (CCA settlers), on aquarium walls (aquarium settlers) and larvae with *Macroblastum dendrospermum* and *Lithophyllum stictiforme* after each experimental treatment. CTRL = normal/control, T1 = acidification and warming, T2 = acidification and warming followed by a heatwave event, SCCA = settler on CCA, SA = settler on aquarium, L = larva.

A total of 46 ASVs were shared by the settlers and larvae with the CCA (Figure 9, Table S12). Of these, 84% were among the 20 most abundant ASVs, contributing between 1.7% and 10.9% and between 0.6% and 14.3% to the total bacterial community in the presence of *M. dendrospermum* and *L. stictiforme*, respectively. Additionally, 22 of these ASVs were part of the core microbiomes of CCA and their related settlers (Figure 9, Table S12).

## 4 Discussion

### 4.1 Climate Change Effects on the Interactions Between CCA and *Eunicella Singularis* Larvae

We show that CCA increase their facilitating role in *Eunicella singularis* larval settlement under future warming and acidification, and we highlight some factors that could underlie this facilitation. The settlement of *E. singularis* larvae was up to three times higher in the presence of CCA exposed to acidification and warming than on unstressed CCA (i.e., under control conditions). Furthermore, we show that the settlement‐inducing capacity of *Macroblastum dendrospermum* is comparable to that of *Lithophyllum stictiforme* (Zelli et al. 2020). We found that for both CCA species subjected to the two treatment conditions, larvae began to settle and metamorphose preferentially on their surface after only 7 to 9 days of pelagic larval duration (PLD). Padrón et al. (2018) recorded a similar PLD (7 to 14 days) for this species in the Gulf of Lion (North Western Mediterranean Sea) and estimated it to be short enough to minimize larval predation while still long enough to ensure connectivity between the fragmented rocky substrates. Thus, the PLD resulting from the CCA facilitation could benefit *E. singularis* recruitment success. In contrast, in the absence of the two CCA (i.e., bare rock), larval settlement started later, after approximately 30 days. Such an extended PLD would likely increase the risk of predation or natural mortality due to the exhaustion of their energetic resources.

In tropical habitats, the capacity of CCA to attract coral larvae also persists under warming and acidification conditions (e.g., Sellares‐Blasco et al. 2023; Sneed et al. 2025). In our study, we report a significant increase in *E. singularis* larval settlement under climate change conditions. Previously, Viladrich et al. (2022) reported successful settlement of larvae on CCA during an experimental heatwave, but the mechanisms promoting these interactions were not explored. By separating the effects of climate change on CCA bacterial communities and exudates production from the larval settlement process, we are able to formulate hypotheses on the potential mechanisms enhancing larval settlement in the presence of the CCA. Furthermore, the gathered empirical knowledge on the capacity of gorgonian larvae to maintain and even increase settlement preferences toward CCA affected by climate change conditions provides valuable information for coral conservation and restoration strategies.

Notably, we measured higher levels of monosaccharides in both CCA species' exudates when they were exposed to the temperature and pH expected by the 21st century SSP5–8.5 scenario followed by a heatwave. The heteropolysaccharide matrix of the cell wall of CCA (e.g., agars and carrageenans) also contains monosaccharides that can be exuded by the algae (Bilan and Usov 2001; Nelson et al. 2013; Cárdenas et al. 2018). Such exudates have already been hypothesized to induce the settlement of tropical coral larvae (Morse and Morse 1991; Tebben et al. 2015). CCA under warming and acidification conditions can increase the production of sugars through the interplay of calcification–decalcification processes of their cell wall (Bergstrom et al. 2023). Such processes strongly depend on the species (Martin and Gattuso 2009; Martin et al. 2013; Bergstrom et al. 2023). We can thus hypothesize that the monosaccharides exudated by the algae increased in response to climate change and could act as chemical cues for *E. singularis* larvae settlement.

On the basis of our results, we also hypothesized a possible role of specific bacterial taxa in the mechanisms described above. Indeed, the heteropolysaccharides of CCA are composed of sulfated monosaccharides (Bilan and Usov 2001), which are known to be used by bacteria belonging to the family Pirellulaceae, particularly the genera *Blastopirellula* and Pir4 lineage, thanks to their sulfatase enzymes (Bondoso et al. 2017; Bergstrom et al. 2023). We found that these genera are among the most abundant in the microbiome of both CCA, along with other core taxa such as NB1‐j group and the genus *Woeseia* (see also Manea et al. 2025), and across treatments, with some of its individuals enriching the stressed *L. stictiforme* microbiomes. Based on these results, we hypothesize that an increased monosaccharides production by *M. dendrospermum* and *L. stictiforme*, in combination with the breakdown of algal polysaccharides by bacteria of the family Pirellulaceae, caused increased sugar exudation and higher attractiveness of CCA toward larvae. As these bacteria taxa are normally associated with CCA, we also hypothesize that CCA polysaccharides' degradation and sugars' exudation are mechanisms that favor *E. singularis* larvae settlement on unstressed CCA. A dose–response experiment should be conducted to confirm the inducing effect of monosaccharides on *E. singularis* larval settlement. Furthermore, we observed a significantly higher abundance of bacteria belonging to the JTB23 clade (class Gammaproteobacteria, ASV806), which is suggested to be a larval settlement inducer for the tropical coral *Acropora tenuis* (Turnlund et al. 2023). These bacteria could thus also have a role in enhancing algal attractiveness in Mediterranean CCA.

Despite a persistent core microbiome, i.e., the consistent presence of shared bacterial taxa (Custer et al. 2023), across treatments, CCA bacterial communities underwent significant changes, especially under T2 treatment (climate change and heatwave). For instance, in both stressed CCA species, we found a significant increase of bacteria belonging to the family Methyloligellaceae (Alphaproteobacteria order Rhizobiales, ASV676), which have been described to live associated with macroalgae by benefiting from the breakdown of algal polymers (Kopprio et al. 2021). These bacteria may have benefited from the polysaccharides' breakdown possibly mediated by Pirellulaceae. In addition, the OM190 group increased in relative abundance under T2 treatment. Members of this group are associated with macroalgae and produce antimicrobial compounds (Pushpakumara et al. 2023). In *L. stictiforme* there was an increase in abundance of the genus SM1A02, found to be thermotolerant (Díaz‐Almeyda et al. 2022). The increase of these taxa might enhance the resistance of the CCA holobiont to stressful conditions. However, in *M. dendrospermum*, we found an increase of the Thermoanaerobaculaceae subgroup, likely opportunistic, because it is commonly associated with acidified environmental conditions (Tangherlini et al. 2021). In the same CCA species, the Sva0996 genus decreased after stressful conditions. This genus, mainly known as a marine nitrogen cycler (Traving et al. 2021), has been recently found to be part of the core microbiome of five different macroalgae species (Brunet et al. 2025). This finding suggests a key role of this genus in macroalgal metabolism, and its decrease in *M. dendrospermum* might negatively affect the CCA. Given the significant impact of warming and acidification on CCA holobionts, studies on bacterial functional traits and their contribution to the host physiology, resilience and ecological functioning, as well as on the long‐term effects of microbiome shifts on CCA metabolism, will be critical. Such studies would allow us to assess the effects of climate change not only on the survival of CCA and on the larva‐CCA interactions but also on gorgonian post‐settlement survival.

### 4.2 Potential Bacteria Acquisition From CCA by *Eunicella Singularis* Early‐Life Stages

Our study provides hints on the influence of CCA on *E. singularis* microbiome development and bacteria acquisition modes. We found significant differences in bacterial community composition at the ASV level driven by holobiont type rather than experimental conditions when comparing larvae, settlers and CCA. This result is in line with studies that observed how host‐associated microbial communities, including for corals, change in composition during host development (Zhou et al. 2017; Angthong et al. 2023; Hochart et al. 2024). We also found that individuals settled on CCA have bacterial communities that are different and richer than those of individuals settled on the aquarium walls and larvae. Similar results were obtained by Hochart et al. (2024), who concluded that the microbial community of coral recruits varied depending on the substrate they grew on. These findings hint at an enrichment of the diversity of the bacterial community of the gorgonian settlers that are in direct contact with the algal holobionts.

We found that most of the ASVs shared between CCA, larvae and settlers were the most abundant in the bacterial communities of the latter two. We also found some ASVs exclusively shared between settlers and the CCA to which they were attached, which suggests that these bacteria are acquired horizontally, potentially from the algae. Corals, as many other holobionts, can acquire microbes not only vertically from parents, but also horizontally from the environment during their life cycle (van Oppen and Blackall 2019). Some of these shared ASVs are typically associated with CCA. For instance, settlers on *L. stictiforme* exposed to both climate change treatments shared bacteria of the genus *Breoghania* (ASV4, Alphaproteobacteria of the family Stappiaceae), previously found associated with tropical CCA exposed to warming conditions (Webster et al. 2011). Bacteria of the genus *Ruegeria* (ASV7, Alphaproteobacteria of the family Rhodobacteraceae) were shared between control *M. dendrospermum* and related settlers. These bacteria have already been found associated with the CCA *Neogoniolithon brassica‐florida* from the same sampling location as this study (i.e., Banyuls‐sur‐Mer). Interestingly, the CCA *L. stictiforme* shared ASV252 with its associated settlers, which belongs to the genus *Aquimarina* (class Bacteroidia), one of the most common bacteria found in *E. singularis* adults (van de Water et al. 2017), but also associated with diverse marine organisms (e.g., Flemer et al. 2012; Nedashkovskaya et al. 2018). We cannot compare this ASV with previous studies on the microbiome of adult gorgonians because of the different 16S rDNA regions used. As such, an ad hoc study would be needed to confirm the transmission of this bacterial taxon from the CCA to the *E. singularis* , and therefore a further key role of CCA for this holobiont beyond the settlement of its larvae.

At the class level, individuals belonging to Alpha and Gammaproteobacteria predominated in all larvae and settlers, regardless of the algae they were in contact with in the aquarium. In most of them, we also found predominant members of the orders Spirochaetales (class Spirochaetia) and Rickettsiales (Alphaproteobacteria). Similarly to the class Bacteroidia, these taxa are also normally found in the core microbiome of adults of *E. singularis* (van de Water et al. 2018). Despite the presence of ASVs belonging to Spirochaetales and Rickettsiales in both CCA, none of them were shared with the larvae and settlers, which might acquire these taxa through their parents (i.e., vertically).

Finally, we found two *Endozoicomonas* ASVs (ASV1070 and ASV5760) in the bacterial communities of two larvae and in several settlers, but they were not abundant. In *E. singularis* adults, bacteria of the genus *Endozoicomonas* dominate the bacterial communities, with a relative abundance between 50% and 80% (van de Water et al. 2017, 2018). These bacteria have been suggested to play key roles in the metabolism and fitness of their host (Hochart et al. 2023; Pogoreutz and Ziegler 2024). Since *Endozoicomonas* were not found associated with all larvae, but they were more prevalent in the settlers, we suggest that they are acquired from the water (i.e., horizontally).

## 5 Conclusions

Here we showed that the settlement of *Eunicella singularis* larvae can be enhanced by two CCA species (*Macroblastum dendrospermum* and *Lithophyllum stictiforme*), especially after they have been exposed to warming and acidification. We also showed that the interaction between CCA and gorgonian settlers may influence gorgonian microbiome composition, regardless of the climate change conditions, with CCA potentially transmitting some bacteria that characterize *E. singularis* adult holobionts.

Combining our results with previous knowledge on the tolerance of both *E. singularis* adults and larvae to climate‐induced stressors (Ferrier‐Pagès et al. 2009; Viladrich et al. 2022), we suggest that this species has a high resilience potential under future climate conditions. Future studies should explore the ability of both *M*. *dendrospermum* and *L. stictiforme*, and the white gorgonian settlers, to survive, grow and reproduce under long‐term climate change conditions.

Overall, we suggest that conservation and restoration strategies for Mediterranean gorgonians should integrate knowledge on CCA‐ *E. singularis* interactions and move beyond single‐species approaches or adults' transplantation toward strategies that foster and maintain positive interspecific interactions across biological levels (from micro to macroorganisms).

## Author Contributions

**E. Manea:** conceptualization, investigation, validation, formal analysis, visualization, data curation, writing – original draft preparation, funding acquisition. **P. E. Galand:** supervision, validation, writing – review and editing. **S. Comeau:** validation, resources, writing – review and editing. **C. Ferrier‐Pagès:** validation, resources, writing – review and editing. **B. Giordano:** investigation, visualization, writing – review and editing. **L. Pezzolesi:** formal analysis, resources, validation, visualization, data curation, writing – review and editing. **J.‐B. Raina:** formal analysis, resources, writing – review and editing. **S. N. Elahee Doomun:** formal analysis, editing. **R. Tignat‐Perrier:** formal analysis, writing – review and editing. **L. Bramanti:** conceptualization, supervision, investigation, validation, formal analysis, data curation, writing – review and editing, funding acquisition.

## Funding

This work was supported by the HORIZON European Union programme Marie‐Sklodowska Curie Actions, 101062275. Prince Albert II of Monaco Foundation; Scholarship from the University of Cagliari; COST Action MAFWORLD, CA20102.

## Ethics Statement

The content and authorship of the submitted manuscript have been approved by all authors, and that all prevailing local, national, and international regulations and conventions, and normal scientific ethical practices, have been respected.

## Conflicts of Interest

The authors declare no conflicts of interest.

## Supporting information


**Data S1:** emi70217‐sup‐0001‐Supinfo1.docx.

**Data S2:** emi70217‐sup‐0002‐Supinfo2.xlsx.

## Data Availability

The bacteria raw amplicon sequences and accompanying metadata have been deposited in the National Center for Biotechnology Information Sequence Read Archive under the project number PRJNA1254713, https://www.ncbi.nlm.nih.gov/sra/PRJNA1254713. The crustose coralline algae sequences have been deposited in the National Center for Biotechnology Information Sequence Read Archive with the accession numbers PV589095–PV589112. The raw metabolome data files were deposited in MetaboLights under accession number MTBLS13051 and are available also at DOI https://doi.org/10.5281/zenodo.15635084.
