# Supplementary material for: Positive Interactions Under Ocean Warming and Acidification: Crustose Coralline Algae Holobionts Enhance Gorgonian Larval Settlement Under Climate Change
Source: Environ Microbiol. 2025 Dec 14;27(12):e70217. doi: 10.1111/1462-2920.70217 (PMC12702612; doi:10.1111/1462-2920.70217)
Supplement: Supplementary file 1 — Data S1: emi70217‐sup‐0001‐Supinfo1.docx. [file EMI-27-e70217-s001.docx]

Supplementary material

**Positive interactions under ocean warming and acidification: crustose coralline algae holobionts enhance gorgonian larval settlement under climate change**

Manea, E.^1^*; Galand, P.E.^1^; Comeau, S.^2^; Ferrier-Pagès, C.^3^; Giordano, B.^1,4^; Pezzolesi, L.^5,6^; Raina, J-B.^7^; Elahee Doomun, S.N.^8^; Tignat-Perrier, R.^3^; Bramanti, L.^1^

^1^Laboratoire d’Ecogeochimie des Environnements Benthiques, LECOB, Observatoire Océanologique de Banyuls sur Mer Centre National de la Recherche Scientifique (CNRS)-Sorbonne Université, Banyuls sur Mer, France

^2^Laboratoire d’Océanographie de Villefranche (LOV), CNRS, Sorbonne université, 06230 Villefranche-sur-Mer, France

^3^Centre Scientifique de Monaco, Coral Ecophysiology team and Unité CSM-Chanel sur les Coraux Précieux, 8 Quai Antoine 1er, MC-98000 Monaco

^4^Department of Life and Environmental Sciences, University of Cagliari, Cagliari, Italy

^5^Department of Biological, Geological and Environmental Sciences (BiGeA), University of Bologna, Via Sant’Alberto 163, 48123 Ravenna, Italy

^6^Interdepartmental Centre for Industrial Research in Renewable Resources, Environment, Sea and Energy (CIRI-FRAME), University of Bologna, Via Sant’Alberto 163, 48123 Ravenna, Italy

^7^PSL Université Paris: EPHE-UPVD-CNRS, USR 3278 CRIOBE, Université de

Perpignan, 52 Avenue Paul Alduy, 66860 Perpignan CEDEX, France

^8^Metabolomics Australia, Bio21 Molecular Science and Biotechnology Institute, University of Melbourne, Parkville, VIC, Australia

*Corresponding author: [elisabetta.manea@obs-banyuls.fr](mailto:elisabetta.manea@obs-banyuls.fr)

Supplementary text related to the Material and Methods

**Experimental setup and treatments**

The acclimatization of CCA thalli was done in an open-circuit aquarium that received a continuous water flow of 1 L min^-1^. The seawater was pumped from Banyuls-sur-Mer Bay at 14 m depth, filtered at 5 µm, and maintain at ≈16.1°C, the temperature of the sampling site. A Sera LED cool daylight 1420 mm / 27 W was adopted to maintain a daily irradiance of 3 µmol photons m^-2^ s^-1^ under a cycle of 10-h light-14-h dark. After acclimatization, fragments of nine thalli of each CCA species (surface = 12.6±2.5 cm^2^) were coated on the underside with non-toxic epoxy glue (Holdfast Epoxy, Aquarium Systems) to avoid possible interactions between *Eunicella singularis* larvae and the organisms colonizing the underside of the CCAs. Each fragment was placed in a 1-liter cylindrical open-circuit aquarium and was maintained in three different treatments for 56 days prior to the introduction of the larvae. The treatments were the following: **Control** (**CTRL**) representing the ambient conditions of pH (pH_T_ = 8.1) and temperature (T ≈ 16.1 °C); **T1** representing the acidified and warmer conditions projected in the SSP5-8.5 scenario for 2100 with pH_T_ value corresponding to 7.70 (Kwiatkowski et al., 2020) and a temperature increase of 2.5 °C (Reale et al., 2022); **T2** corresponding to the same conditions than T1 (pH ≈ 7.70, T = + 2.5 °C) for 35 days, followed by a 21-day heatwave event at 26 °C representative of the projections in the study area starting from 2050 (Galli et al., 2017). The 26 °C temperature was achieved over 10 days with a daily increase of 0.5°C. Temperature and pH conditions were controlled as described below.

The same cylinders were used to test for the larval settlement with three replicated cylinders used for each algal species and treatment (18 cylinders in total). A total of six cylinders (three replicates for the two algal species) were used for each environmental condition and were arranged in three larger aquaria (later referred to as “water bath”), each dedicated to one of the experimental treatments (T1, T2, CTRL). In addition, three cylinders containing only bare rocks, which were first combusted at 459 °C for four hours (to remove organisms and organic matter) and coated with epoxy on one side, were added to the CTRL treatment aquarium. The remaining fragments of the CCA thalli that were not put in the cylinders were placed in their corresponding water bath. These CCA fragments were used to characterize the composition of the CCA bacterial communities after treatments, just before the addition of the larvae to the cylinders. Finally, three intact unfragmented thalli for each CCA species were kept only in the T2 water bath to characterize exo-metabolite profiles after treatment. Seawater pumped from Banyuls-sur-Mer Bay at 14 m depth and filtered at 5 um was continuously flowing into four 100-liter tanks, the header tanks. These tanks supplied the 21 cylinders at a rate of 50 mL min^-1^ which subsequently fed the three water baths.

During the 56 days of treatment, in two out of the four header tanks, pH and temperature were controlled using dedicated controllers and probes with the APEX Neptune Systems ([www.neptunesystems.com](http://www.neptunesystems.com)). The header tanks randomly supplied the 12 cylinders dedicated to T1 and T2 treatments. The temperature was adjusted daily to mimic its natural seasonal daily variation. In the other two 100-litre tanks, temperature and pH were those of the natural seawater, and they randomly supplied the nine CTRL cylinders. The heatwave event of T2 treatment was simulated by increasing the seawater temperature specifically in the water bath containing the six dedicated cylinders with dedicated temperature controller and probe. After 56 days of treatment, temperature and pH were maintained at ambient conditions. The Sera LED cool daylight 1420 mm / 27 W was adopted to maintain a daily irradiance of 3 µmol photons m^-2^ s^-1^ under a cycle of 10 to 12-h light and, respectively, 14 to 12-h dark following the monthly changes.

Temperature and pH in all experimental aquaria and cylinders were measured weekly using a handheld pH meter (IP67 3110, Fisher Scientific) calibrated with a seawater pH TRIS buffer (batch no. T33 provided by Andrew G. Dickson, Scripps Institution of Oceanography, USA) before each set of measurements. Salinity data during the experiment were obtained from measurements performed in the bay of Banyuls. To confirm that total alkalinity was not altered by metabolic activity, it was measured biweekly in each experimental condition by potentiometric titration using a Metrohm 888 Titrando following the method of Dickson et al. (2007). Titrations of certified reference material (batch no. 186) provided by Andrew G. Dickson were used to assess the accuracy of the measurements and were within 6.5 µmol kg^−1^ of the reference value. pH_T_, temperature, total alkalinity, and salinity were used to calculate the other carbonate chemistry parameters using R package seacarb (Gattuso et al., 2024).

After the 56 days of treatment, the CCA thalli kept in the larger aquaria were sampled and frozen immediately at -80 °C for subsequent analysis of the associated bacterial communities. For the CCA fragments in the cylinders, water conditions in all experimental systems were restored to normal conditions five days before the addition of gorgonian larvae.

**CCA DNA extraction, amplification, sequencing, and phylogenetic analyses**

The taxonomic identification of CCAs was performed as in Manea et al. (2025). The silica-dried CCA samples were pulverized using sterile mortars, and the DNA extraction was performed following the modified protocol of the Qiagen DNeasy Blood & Tissue Kit (Qiagen, Crawley, UK) by Broom et al. (2008). Samples were incubated in 1.5 mL centrifuge tubes with 400 µL of extraction buffer ATL and 25 mAU of proteinase K at 65 °C for 4 h. Samples were subsequently incubated at 70 °C for 10 minutes after the addition of 400 µL of lysis buffer AL. Samples were then centrifuged at full speed in a benchtop centrifuge for 10 minutes. After supernatants transfer to new 1.5 mL centrifuge tubes, 400 µL of ethanol (96-100%) were added. Once mixed, the obtained solution was transferred to a DNeasy Mini spin column in a 2 mL collection tube, and then processed following the manufacturer instructions.

The *psb*A gene was PCR-amplified according to Pezzolesi et al. (2017, 2019), while the mitochondrial COI-5P fragment was PCR-amplified in *M.* *dendrospermum* following Peña et al. (2015). PCR products were visualized in 1.5% agarose gels stained with Midori Green using Low DNA Mass Ladder (Invitrogen, Carlsbad, CA, USA) as a reference, and PCR products with expected lengths and yields were purified and sequenced by BMR Genomics (Padua, Italy).

The quality of the sequences was assessed by visual inspection of the electropherograms using the Chromas software (Version 2.6.6; Technelysium Pty LTD, South Brisbane, Australia). Alignment was performed using ClustalW and default settings and phylogeny constructed by means of MEGA software (Version 11.0.13, [www.megasoftware.net](http://www.megasoftware.net); Tamura et al., 2021). In addition, NCBI database ([www.ncbi.nlm.nih.gov](http://www.ncbi.nlm.nih.gov)) was searched for publicly available sequences of *Lithophyllum stictiforme* complex and *Mesophyllum* genus (as *Mesophyllum macroblastum* is now recognized as *Macroblastum dendrospermum,* Athanasiadis & Ballantine, 2024), and some representatives were selected to be included in the alignments, as in Manea et. (2025). The alignment was assembled including the *psb*A sequences of *Lithophyllum* *dentatum* (Kützing) Foslie*, L. hibernicum* Foslie*,* and *L. bathyporum* Athanasiadis & D.L.Ballantine, and psbA sequences of the Melobesioideae *Phymatolithon calcareum* (Pallas) W.H. Adey & McKibbin and *Lithothamnion corallioides* (P.L. Crouan & H.M. Crouan) P.L. Crouan & H.M. Crouan as outgroups in the respective data sets, according to previous studies (Pezzolesi et al, 2019; Peña et al., 2015; Manea et al., 2025). The sequences’ alignment of *L. stictiforme* *psbA* sequences was 816 base pairs long, while those of *M. dendrospermum* ranged from 599 to 851 base pairs. The COI-5P of *M. dendrospermum* alignment resulted in 608 bp. Neighbor-Joining (NJ) distance analyses were performed on all data sets using Maximum Composite Likelihood model in MEGA software, with nodal support assessed by 1,000 bootstrap (BR) resamplings. Phylogenetic relationships were inferred using Maximum likelihood (ML) analyses in MEGA software, under a generalized time-reversible with gamma+invariant sites heterogeneity model (GTR+G+I), and a generalized time-reversible gamma distributed (GTR+G) alignments, for the COI-5P and *psbA* alignments, respectively. The bootstrap consisted of 1000 replicates with complete deletion option, i.e. eliminating positions containing gaps and missing data (Saitou & Nei, 1987; Nei & Kumar, 2000; Pezzolesi et al., 2019; Manea et al., 2025).

**Bacteria DNA amplification and sequencing from *E. singularis* larvae and settlers**

Bacteria DNA amplification from single larvae and settlers was carried out using a direct PCR approach as described in Tignat-Perrier et al. (2025) to avoid losing the small quantity of bacteria DNA associated with the samples. Samples were incubated for 20 min at 65 °C in extraction buffer (qPCR ProbesMaster kit, Jena Bioscience) supplemented with 0.1 µL of Proteinase K solution (600 U/mL; Qiagen) for cell lysis and DNA release. The V3-V4 region of the 16S rRNA gene was then amplified in three replicates per sample using the direct qPCR ProbesMaster kit (Jena Bioscience) and the forward and reverse primers 341F 5′-CCTACGGGNGGCWGCAG-3′ and 785R 5′-GACTACHVGGGTATCTAATCC-3′, respectively (Klindworth et al., 2013). The reaction mixture contained 25 µL of direct qPCR ProbesMaster mix, 0.75 µL of each primer (10 µM), 1 µL of sample (in extraction buffer) and RNAse-free water to complete the final 50 µL volume. The PCR 3-steps program consisted of an initial step at 95 °C for 5 min for enzyme activation, 40 cycles of 15 sec at 95 °C, 30 sec at 55 °C and 30 sec at 60 °C for denaturation, hybridization and elongation, respectively, then a final step at 60 °C for 10 min. Negative controls (i.e., PCR without sample material) were processed at the same time as the samples. The three PCR reactions per sample were pooled and amplicons were purified using AMPure XP beads (Beckman Coulter). Amplification was checked using the Bioanalyzer DNA 1000 kit (Agilent), and samples showing amplification were sent for library preparation and sequencing following Illumina’s standard “16S Metagenomic Sequencing Library Preparation” protocol (Illumina, 2013).

**CCA metabolite sampling, extraction and composition**

Samples for CCAs’ exo-metabolite characterization were collected in aquarium after T2 treatment (i.e., warming and acidification followed by heatwave). Additional six samples (three for each CCA species) were collected in situ and used as controls for comparison. In both cases, a volume of 200 μL of seawater was collected by using sterile syringes on the surface of each CCA thallus, for a total of 12 samples (six in situ samples, hereafter referred as “in situ”, and six samples in T2 treatment aquarium). The samples were immediately filtered through a sterilized 0.2 μm filter (Minisart Syringe Filter, diameter 47 mm, Sartorius) and frozen at -80°C until metabolites extraction.

Samples were subjected to a solid-phase extraction (SPE) using Strata-X 33 µm Polymeric Reversed Phase columns (Phenomenex SAS, US) following a protocol adapted from Dittmar et al. (2008). Specifically, samples were acidified to pH 2 using 10% HCl (made with HPLC-water from HCl puriss. 32%, Fluka, Sigma), and metabolites were adsorbed onto the SPE cartridges using gravity flow not exceeding a flow rate of 1 mL min^-1^. SPE cartridges were then dried for 20 min before being eluted with 80% HPLC-grade methanol and then dried using a rotary evaporator. The dried extracts were reconstituted in 200 ul 3:1 methanol/Milli-Q water with 0.4 nmol of ^13^C_5_^15^N valine and ^13^C_6_ sorbitol. The samples were sonicated for 20 min at 4°C, then centrifuged at 4°C for 20 min at 16000 *g* using an Eppendorf centrifuge 5430 R. Seventy-five μL of the supernatant was dried for the GC-MS analysis.

Dried samples for targeted analysis were prepared by adding 25 μL of methoxyamine hydrochloride (30 mg ml^−1^ in pyridine) followed by shaking at 37 °C for 2 h. Samples were then derivatized with 25 μL of N,O-bis (trimethylsilyl)trifluoroacetamide with trimethylchlorosilane (BSTFA with 1% TMCS, Thermo Scientific) for 1 h at 37 °C. Samples were left for 1 h before 1 μL was injected onto the gas chromatography column using a hot needle technique.

The GC–MS system used was composed of an AOC6000 autosampler, a 2030 Shimadzu gas chromatograph and a TQ8050 quadrupole mass spectrometer (Shimadzu). The mass spectrometer was tuned according to the manufacturer’s recommendations using tris-(perfluorobutyl)-amine (CF43). GC–MS was performed on a 30 m Agilent DB-5 column with 1 μm film thickness and 0.25 mm internal diameter column. The injection temperature (inlet) was set at 280 °C, the MS transfer line at 280 °C and the ion source adjusted to 200 °C. Helium was used as the carrier gas at a flow rate of 1 mL min^−1^, and argon gas was used as the collision cell gas to generate the multiple reaction monitoring (MRM) product ion. Sample analysis was performed under the following temperature program; start at injection 100 °C, a hold for 4 min, followed by a 10 °C min^−1^ oven temperature ramp to 320 °C following final hold-off for 11 min. Approximately 520 quantifying MRM targets were collected using Shimadzu Smart Database along with a qualifier for each target that covers about 350 endogenous metabolites and multiple ^13^C-labelled internal standards. Both chromatograms and MRMs were evaluated using the Shimadzu GCMS LabSolutions Insight software (version 3.6). Resulting area responses were normalized to the internal standard ^13^C_6_-sorbitol area response.

**Data analysis and statistics linked to the larvae settlement experiment**

**Effect of substrate and treatment on larval settlement**

To assess the effects of substrate type and experimental treatments on *Eunicella singularis* larval settlement, we performed two separate statistical analyses. This approach was necessary due to differences in substrate characteristics and the applicability of the pre-conditioning treatments.

***Effect of Substrate Type***

We first tested the effect of substrate type on larval settlement using a one-way ANOVA. The experimental design included three substrate types: bare rock, and two species of crustose coralline algae (*Lithophyllum stictiforme* and *Macroblastum dendrospermum*). Settlement was measured as the total number of settlement events recorded over a 59-day experimental period during which settlement was recorded daily in three replicate aquaria for each substrate type.

***Effect of Experimental Treatments***

A second analysis was conducted to examine the effects of ocean acidification and marine heat waves on larval settlement, specifically on the two CCA species. Bare rock was excluded from this analysis because the experimental treatments involved a pre-conditioning phase, which aimed to alter the microbial community associated with CCA surfaces. Since bare rock has been previously sterilized, its inclusion in the analysis would have compromised the statistical validity of the results. To test the effects of substrate type (CCA species) and treatment, we employed a two-way ANOVA with Substrate (2 levels: *Lithophyllum stictiforme* and *Macroblastum dendrospermum*) and Treatment (3 levels: CTRL, T1, and T2) as independent factors.

The underlying hypothesis was that experimental treatments can modify CCA properties (microbial community and metabolites produced), potentially altering their capacity to promote larval settlement. By preconditioning the substrates, we aimed to simulate how future climate scenarios might influence the settlement process through changes in the algae/settlement interactions.

**Effect of substrate and treatment on the timing of larval settlement**

To determine whether substrate type or treatment conditions influenced the timing of larval settlement, we used the day of first settlement as the response variable. This analysis was based on the hypothesis that substrate type or treatment conditions could either accelerate or delay larval settlement. The day of first settlement was recorded for each substrate and treatment in the three replicate aquaria. Two separate ANOVA models were applied to analyze the data:

***Effect of Substrate Type***

A one-way ANOVA was performed to assess the effect of substrate type on settlement timing. The experimental design included three substrates: bare rock, *Lithophyllum stictiforme*, and *Macroblastum dendrospermum*.

***Effect of Experimental Treatments***

A two-way ANOVA was conducted to test if settlement timing is influenced by treatment conditions and if this effect varied between substrates. The experimental design included two independent factors: Substrate (2 levels: *Lithophyllum stictiforme* and *Macroblastum* *dendrospermum*) and Treatment (3 levels: CTRL, T1, and T2). In both analyses, the response variable was the day of first settlement, measured as the day in which the first settlement event was observed in each replicate aquarium.

Supplementary Figures


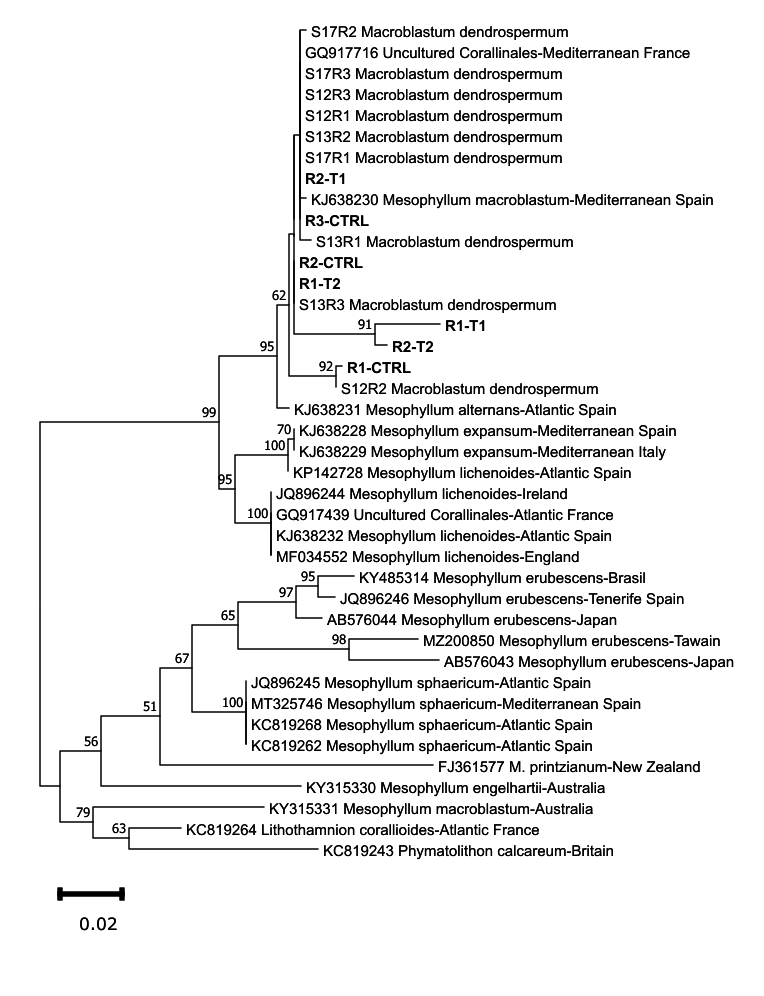


Fig. S1. Phylogenetic tree of *Macroblastum dendrospermum*. Phylogenetic tree inferred from ML analysis of the *psbA* sequences of the Mediterranean *M. dendrospermum* and other publicly available sequences for Mediterranean *Mesophyllum.* Bootstrap ML values > 60% are shown for each node. Members of the subfamily Melobesioideae were used as outgroup. Scale bar: 0.02 substitutions per site. **Sequences generated in the present work are marked in bold.**


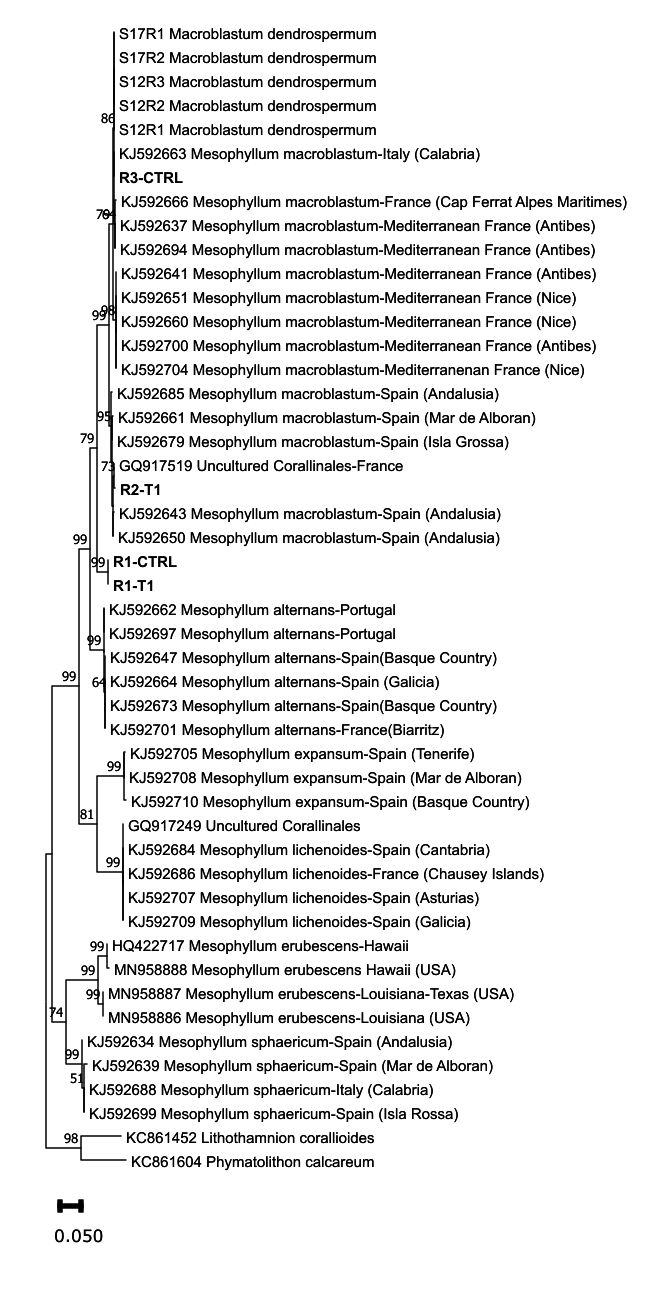


Fig. S2. Phylogenetic tree of *Macroblastum dendrospermum*. Phylogenetic tree inferred from ML analysis of the COI-5P sequences of Mediterranean *M. dendrospermum* and other publicly available sequences for the genus *Mesophyllum*. Bootstrap values > 60% are shown for each node. Members of the subfamily Melobesioideae were used as outgroup. Scale bar: 0.05 substitutions per site. **Sequences generated in the present work are marked in bold.**


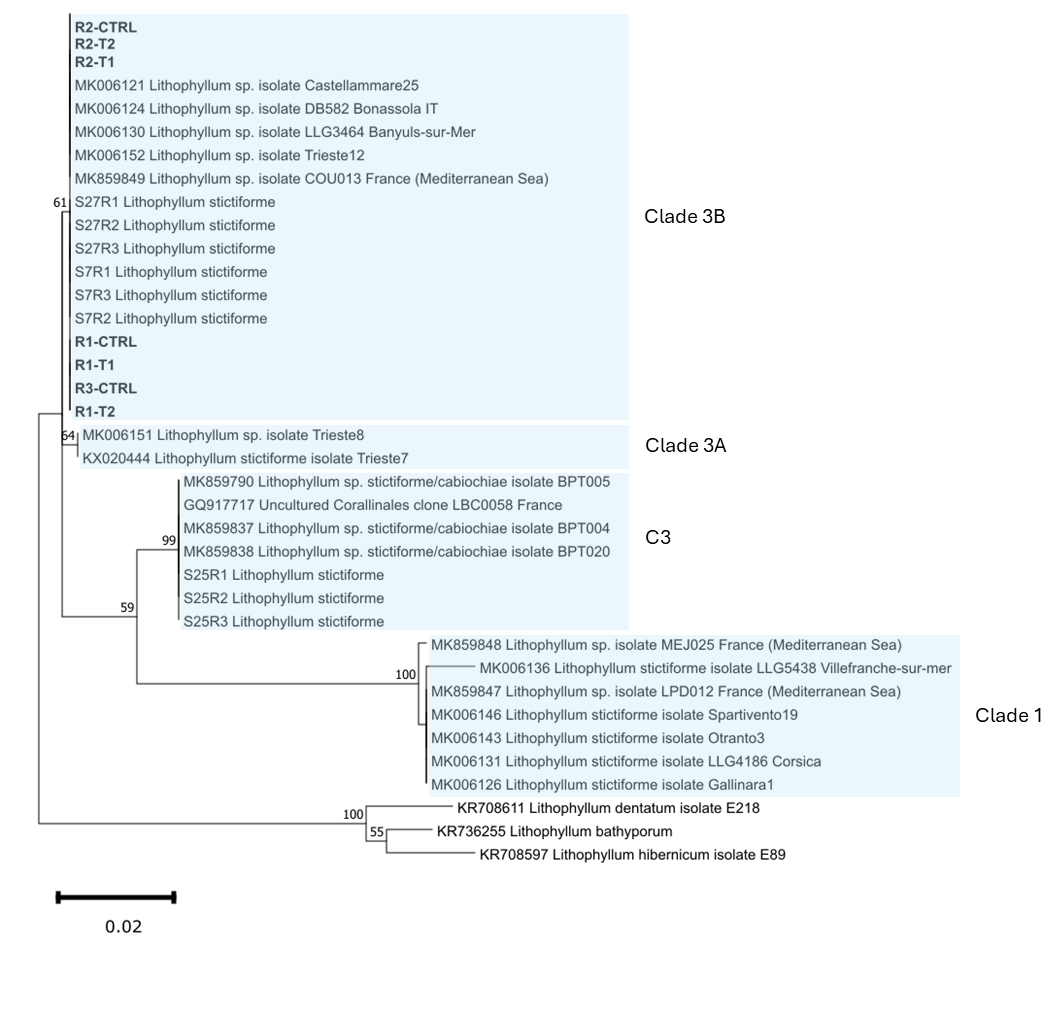


Fig. S3. Phylogenetic tree of *Lithophyllum stictiforme.* Phylogenetic tree inferred from ML analysis of the *psbA* sequences of Mediterranean *L. stictiforme* and other publicly available sequences for this complex. Bootstrap ML values > 60% are shown for each node. *L. dentatum*, *L. hibernicum* and *L. bathyporum* were used as outgroup. Clades refer to the classification provided in previous works (Pezzolesi et al., 2019; De Jode et al., 2019; Manea et al., 2025). Scale bar: 0.02 substitutions per site. **Sequences generated in the present work are marked in bold.**

*
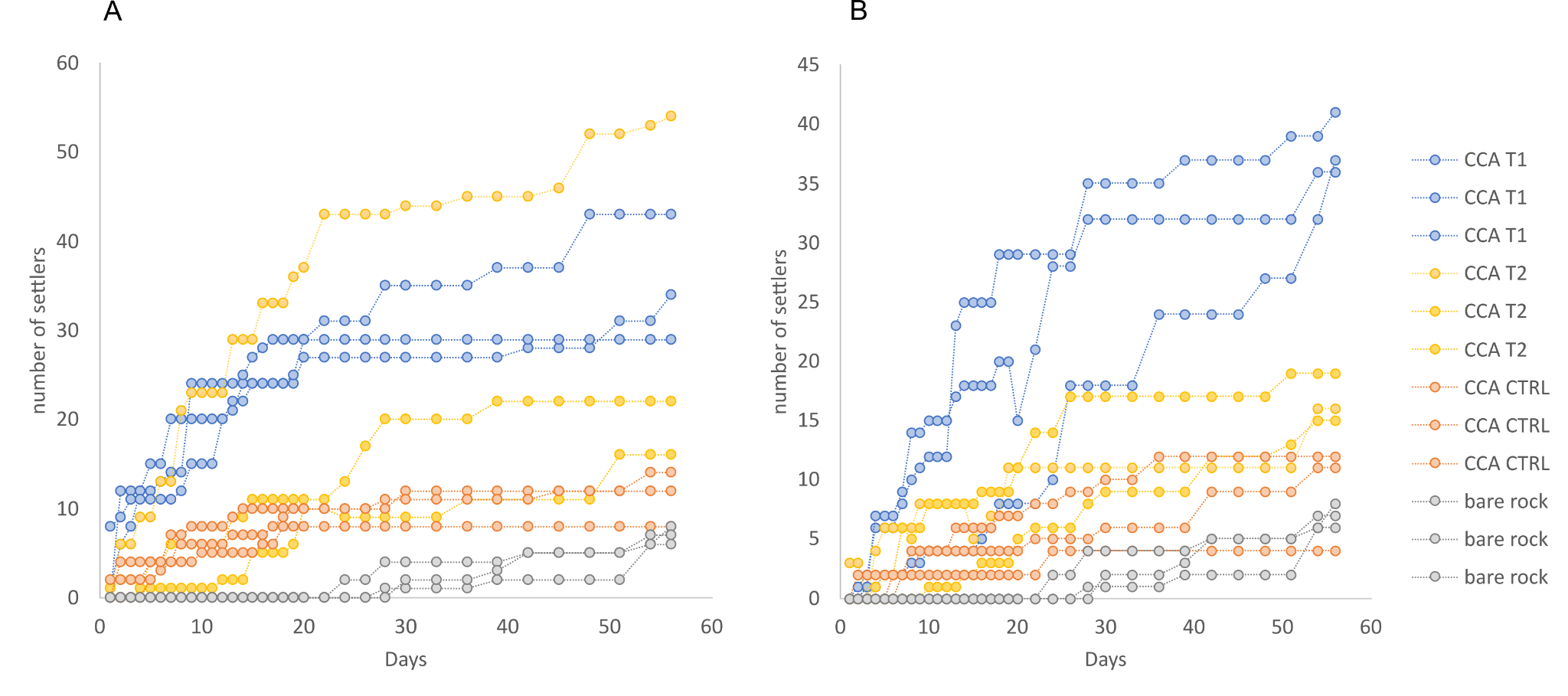
*

Fig. S4. Cumulative number of settlers for each substrate and treatment recorded daily during the settlement experiment (lasted 56 days), in the presence of A) *M. dendrospermum* and B) *L. stictiforme*. CTRL = control (no treatment), T1 = acidification and warming, T2 = acidification and warming followed by a heatwave event, bare rock = control substrate without CCA and without treatment.


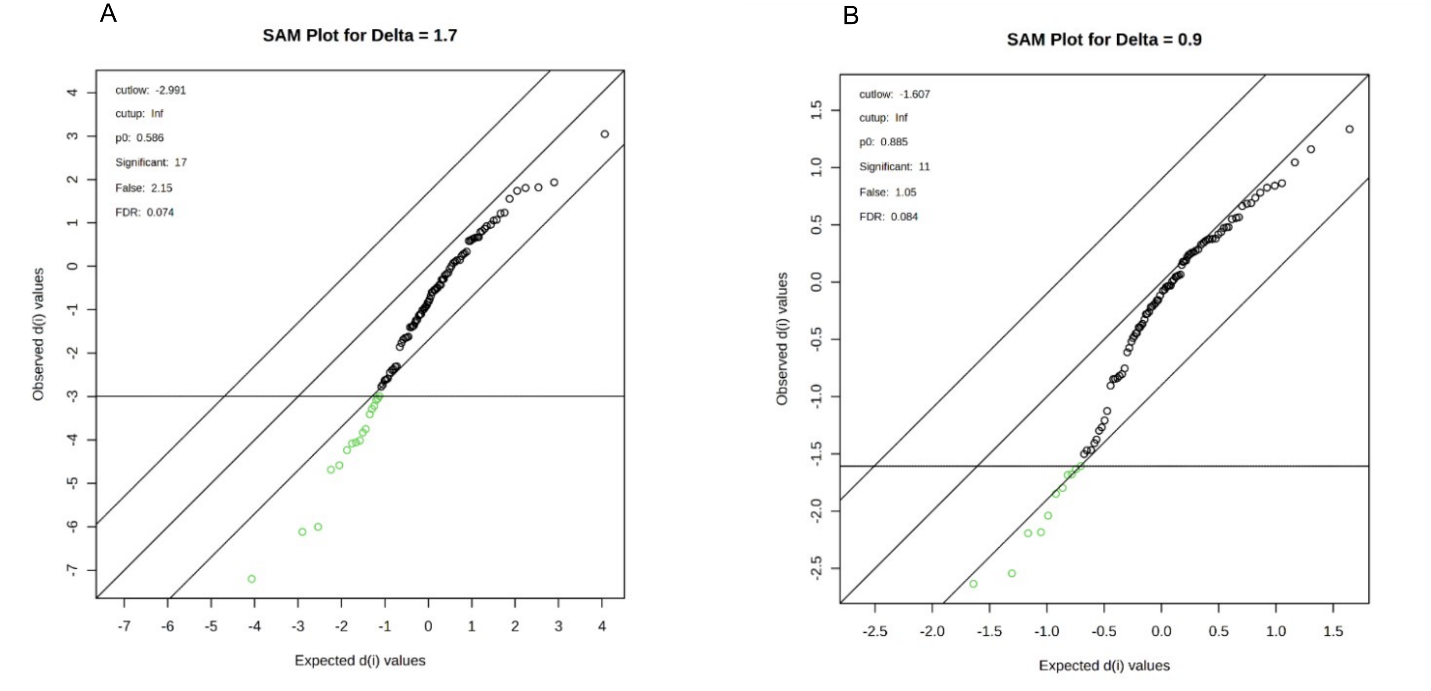


Fig. S5. Output of the multi-class Significance Analysis of Metabolomics SAM (based on F-statistics) for A) *M. dendrospermum* and B) *L. stictiforme*.


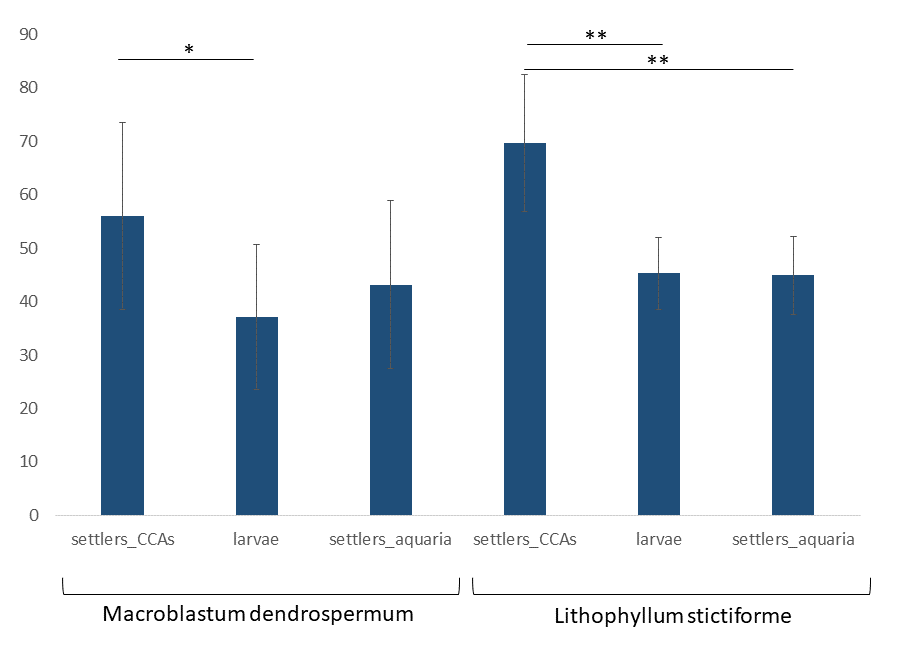


Fig. S6. Bacterial community richness of *E. singularis* larvae and settlers on CCAs and aquarium surface in *M. dendrospermum* and *L. stictiforme* aquaria. *p*-value ≤ 0.5*, *p*-value ≤ 0.01**.


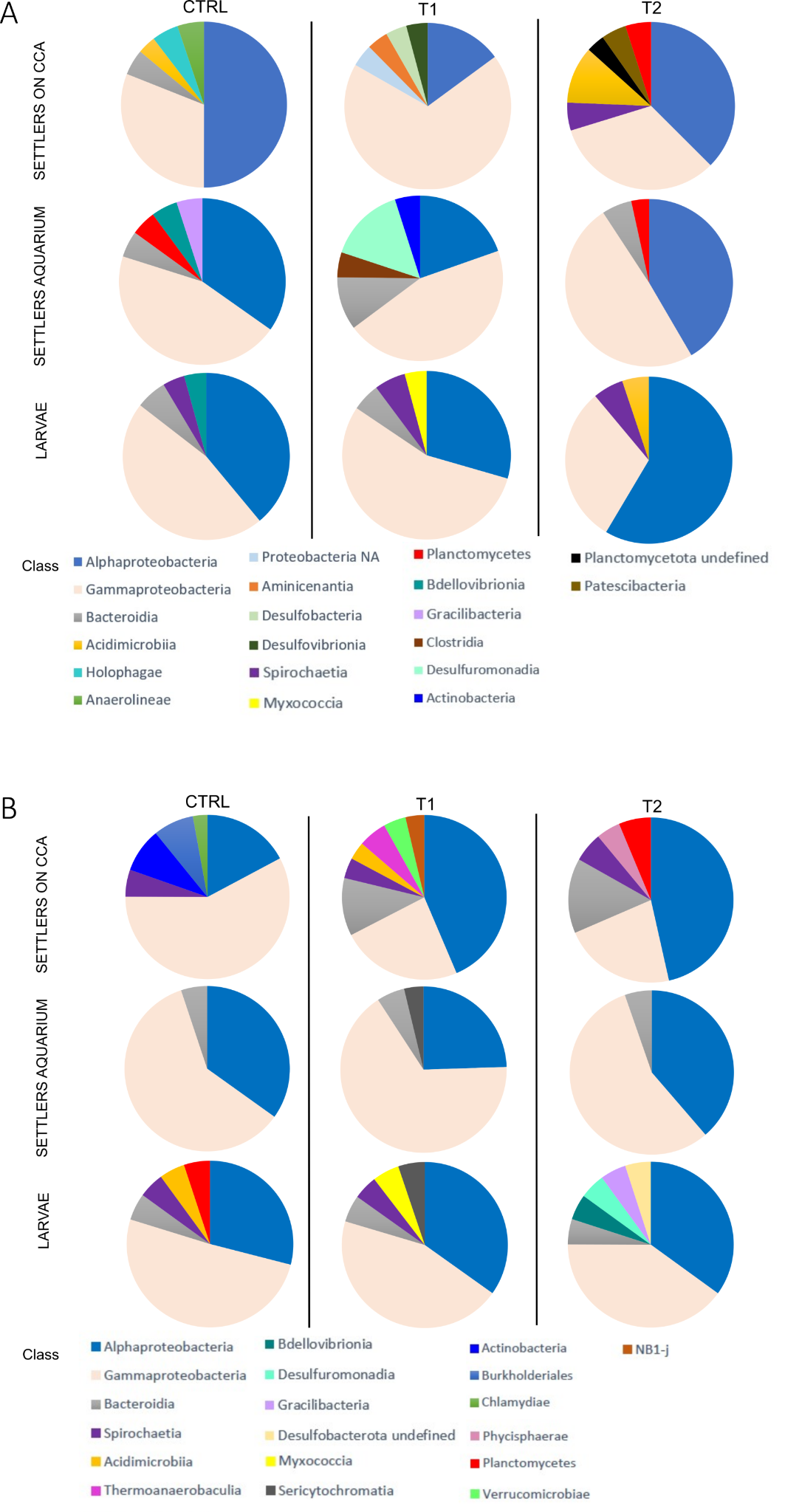


Fig. S7. Twenty ASVs that contributed the most to the microbial community composition of settlers on CCA, larvae, settlers on aquarium surface, in presence of A) *M. dendrospermum* and B) *L. stictiforme* after each experimental treatment. ASVs are grouped at the Class level.

Supplementary Tables

Table S2. Measured and estimated sea water physicochemical parameters of the pH treatments in the experimental header tanks and cylinders for salinity (*S*), temperature (*T*), total alkalinity (*A*_T_), dissolved inorganic carbon (*C*_T_), pH_T_, *p*CO_2_, calcite (Ωc) and aragonite (Ωa) saturation. Values are means ± SD. Calculated concentrations of *C*_T_, *p*CO_2_, Ωc, Ωa are shown.

| Treatment | pH_T_ | *T* (˚C) | *A*_T_  (µmol kg^-1^) | *C*_T_  (µmol kg^-1^) | Salinity | *p*CO_2_ (µatm) | Ωc | Ωa |
| --- | --- | --- | --- | --- | --- | --- | --- | --- |
| CTRL  n=88 | 8.1±0.1 | 16.1±1.2 | 2560.1±15.6 | 2258.3±32.6 | 37.9±0.2 | 372.5±67.3 | 5.1±0.5 | 3.3±0.3 |
| T1  n=70 | 7.7±0.1 | 18.1±0.7 | 2581.3±32.7 | 2478.2±44.5 | 37.9±0.1 | 1204.2±255.5 | 2.3±0.4 | 1.5±0.3 |
| T2  n=21 | 7.7±0.1 | 23.3±3 | 2570.8±6.6 | 2416.6±30 | 37.9±0.2 | 1072.1±248.4 | 3±0.4 | 2±0.3 |

Table S3. Output of the Post hoc pairwise comparisons performed with the Tukey HSD test to test for the effect of the substrate type, bare rock, *M. densdrospermum* and *L. stictiforme*, on *E. singularis* larvae settlement events’ number.

| **Substrates** | **diff** | **lwr** | **upr** | **p adj** |
| --- | --- | --- | --- | --- |
| ***L. stictiforme* – bare rock** | 9.666667 | -1.6916293 | 21.02496 | 0.0886054 |
| ***M. densdrospermum* – bare rock** | 12.333333 | 0.9750374 | 23.69163 | 0.0362924 * |
| ***M. densdrospermum*– *L. stictiforme*** | 2.666667 | -8.6916293 | 14.02496 | 0.7611555 |

Table S4. Output of the Post hoc pairwise comparisons conducted using the Tukey HSD test to test for the effect of treatments on CCA on *E. singularis* larvae settlement events’ number.

| **Treatment** | **diff** | **lwr** | **upr** | **p adj** |
| --- | --- | --- | --- | --- |
| **T1 - CTRL** | 35.50000 | 20.3603254 | 50.639675 | 0.0001157*** |
| **T2 - CTRL** | 15.83333 | 0.6936587 | 30.973008 | 0.0402942* |
| **T2 – T1** | -19.66667 | -34.8063413 | -4.526992 | 0.0120169* |

Table S5. Output of the Post hoc pairwise comparisons performed with the Tukey HSD test to test for the effect of the substrate type, bare rock, *M. densdrospermum* and *L. stictiforme*, on the day of first settlement of *E. singularis* larvae.

| **Substrates** | **diff** | **lwr** | **upr** | **p adj** |
| --- | --- | --- | --- | --- |
| ***L. stictiforme* – bare rock** | -22.333333 | -31.09171 | -13.574956 | 0.0005637*** |
| ***M. densdrospermum* – bare rock** | -24.666667 | -33.42504 | -15.908289 | 0.0003250*** |
| ***M. densdrospermum*– *L. stictiforme*** | -2.333333 | -11.09171 | 6.425044 | 0.7068602 |

Table S6. Output of the PERMANOVA analysis carried out on Bray Curtis matrix of Hellinger transformed ASV data to test for differences in composition of bacterial communities associated with CCAs under different treatments. The analyses were carried out on each CCA species considering experimental treatments (*=p≤0.05, **=p≤0.01, ***=p≤0.001). The outputs of the Betadisper analyses applied to test for data dispersion homogeneity between CCA treatments are also reported.

|  | **PERMANOVA** | | | | | | **BETADISPER** | |
| --- | --- | --- | --- | --- | --- | --- | --- | --- |
| **CCA comparison** | **Source** | **Df** | **SumsOfSqs** | **F.Model** | **R2** | **Pr(>F)** | **F.Model** | **Pr(>F)** |
| Between *M. dendrospermum* thalli under different experimental treatments | Treatment | 2 | 0.72405 | 1.4542 | 0.32648 | 0.0049** | 1.2323 | 0.3561 |
|  | Residuals | 6 | 1.49370 |  | 0.67352 |  |  |  |
|  | Total | 8 | 2.21774 |  | 1 |  |  |  |
| Between *L.stictiforme* thalli under different experimental treatments | Treatment | 2 | 0.92791 | 2.0669 | 0.40792 | 0.0046** | 0.2231 | 0.8064 |
|  | Residuals | 6 | 1.34684 |  | 0.59208 |  |  |  |
|  | Total | 8 | 2.27475 |  | 1 |  |  |  |

Table S11. Output of the pairwise comparison carried out on Bray Curtis matrix of Hellinger transformed ASV data to test for differences in composition of bacterial communities associated to CCAs, larvae, settlers on CCAs, and settlers on aquaria surface.

| ***Macroblastum dendrospermum*** | **Source** | **Df** | **SumsOfSqs** | **F.Model** | **R2** | **Pr(>F)** |
| --- | --- | --- | --- | --- | --- | --- |
| Between larvae and CCA | Model | 1 | 1.9402 | 11.383 | 0.50856 | 0.0014*** |
|  | Residuals | 11 | 1.8749 |  | 0.49144 |  |
|  | Total | 12 | 3.8151 |  | 1 |  |
| Between larvae and settlers on the aquarium | Model | 1 | 0.6069 | 3.3904 | 0.14495 | 0.00006*** |
|  | Residuals | 20 | 3.5799 |  | 0.85505 |  |
|  | Total | 21 | 4.1868 |  | 1 |  |
| Between larvae and settlers on CCA | Model | 1 | 1.5576 | 5.4099 | 0.2129 | 0.00001*** |
|  | Residuals | 20 | 5.7585 |  | 0.7871 |  |
|  | Total | 21 | 7.3161 |  | 1 |  |
| Between settlers on the aquarium and CCA | Model | 1 | 2.0494 | 11.176 | 0.42696 | 0.00009*** |
|  | Residuals | 15 | 2.7506 |  | 0.57304 |  |
|  | Total | 16 | 4.8 |  | 1 |  |
| Between settlers on CCA and CCA | Model | 1 | 1.4646 | 4.457 | 0.22907 | 0.00005*** |
|  | Residuals | 15 | 4.9291 |  | 0.77093 |  |
|  | Total | 16 | 6.3938 |  | 1 |  |
| Between settlers on CCA and settlers on the aquarium | Model | 1 | 1.9304 | 6.983 | 0.2254 | 0.00001*** |
|  | Residuals | 24 | 6.6342 |  | 0.7746 |  |
|  | Total | 25 | 8.5646 |  | 1 |  |
| ***Lithophyllum stictiforme*** |  |  |  |  |  |  |
| Between larvae and CCA | Model | 1 | 1.9521 | 15.049 | 0.60078 | 0.002** |
|  | Residuals | 10 | 1.2972 |  | 0.39922 |  |
|  | Total | 11 | 3.2493 |  | 1 |  |
| Between larvae and settlers on the aquarium | Model | 1 | 0.43677 | 3.4799 | 0.16992 | 0.00007*** |
|  | Residuals | 17 | 2.13369 |  | 0.83008 |  |
|  | Total | 18 | 2.57046 |  | 1 |  |
| Between larvae and settlers on CCA | Model | 1 | 1.5157 | 6.2054 | 0.26741 | 0.00001*** |
|  | Residuals | 17 | 4.1525 |  | 0.73259 |  |
|  | Total | 18 | 5.6682 |  | 1 |  |
| Between settlers on the aquarium and CCA | Model | 1 | 2.0241 | 12.731 | 0.49477 | 0.00009*** |
|  | Residuals | 13 | 2.0669 |  | 0.50523 |  |
|  | Total | 14 | 4.091 |  | 1 |  |
| Between settlers on CCA and CCA | Model | 1 | 1.3075 | 4.1603 | 0.24244 | 0.0012** |
|  | Residuals | 13 | 4.0856 |  | 0.75756 |  |
|  | Total | 14 | 5.3931 |  | 1 |  |
| Between settlers on CCA and settlers on the aquarium | Model | 1 | 1.7234 | 7.0024 | 0.25933 | 0.00001*** |
|  | Residuals | 20 | 4.9222 |  | 0.74067 |  |
|  | Total | 21 | 6.6455 |  | 1 |  |

References

Acosta‐González, A., Rosselló‐Móra, R., & Marqués, S. (2013). Characterization of the anaerobic microbial community in oil‐polluted subtidal sediments: aromatic biodegradation potential after the Prestige oil spill. *Environmental microbiology*, *15*(1), 77-92. doi:10.1111/j.1462-2920.2012.02782.x

Agogué, H., Casamayor, E. O., Bourrain, M., Obernosterer, I., Joux, F., Herndl, G. J., & Lebaron, P. (2005). A survey on bacteria inhabiting the sea surface microlayer of coastal ecosystems. *FEMS microbiology ecology*, *54*(2), 269-280. doi: 10.1016/j.femsec.2005.04.002.

Athanasiadis, A., & Ballantine, D. L. (2024). Anatomy and Classification of the Mesophyllaceae (corallinales, Rhodophyta), Based on Phylogenetic Principles. *Smithsonian Institution Scholarly Press.*

Barco, R. A., Hoffman, C. L., Ramírez, G. A., Toner, B. M., Edwards, K. J., & Sylvan, J. B. (2017). In‐situ incubation of iron‐sulfur mineral reveals a diverse chemolithoautotrophic community and a new biogeochemical role for Thiomicrospira. *Environmental microbiology*, *19*(3), 1322-1337.doi:10.1111/1462-2920.13666.

Bayer, T., Neave, M. J., Alsheikh-Hussain, A., Aranda, M., Yum, L. K., Mincer, T., ... & Voolstra, C. R. (2013). The microbiome of the Red Sea coral *Stylophora pistillata* is dominated by tissue-associated Endozoicomonas bacteria. *Applied and environmental microbiology*, *79*(15), 4759-4762. doi: 10.1128/AEM.00695-13.

Bessette, S., Fagervold, S. K., Romano, C., Martin, D., Bris, N. L., & Galand, P. E. (2014). Diversity of bacterial communities on sunken woods in the Mediterranean Sea. *Journal of Marine Science and Technology*, *22*(1), 7. doi: 10.6119/JMST-013-0829-2

Blanchet, E., Prado, S., Stien, D., Oliveira da Silva, J., Ferandin, Y., Batailler, N., ... & Lami, R. (2017). Quorum sensing and quorum quenching in the Mediterranean seagrass Posidonia oceanica microbiota. *Frontiers in Marine Science*, *4*, 218. [doi.org/10.3389/fmars.2017.00218](https://doi.org/10.3389/fmars.2017.00218)

Broom, J. E., Hart, D. R., Farr, T. J., Nelson, W. A., Neill, K. F., Harvey, A. S., & Woelkerling, W. J. (2008). Utility of psbA and nSSU for phylogenetic reconstruction in the Corallinales based on New Zealand taxa. *Molecular phylogenetics and evolution*, *46*(3), 958-973. doi:10.1016/j.ympev.2007.12.016

Couradeau, E., Roush, D., Guida, B. S., & Garcia-Pichel, F. (2017). Diversity and mineral substrate preference in endolithic microbial communities from marine intertidal outcrops (Isla de Mona, Puerto Rico). *Biogeosciences*, *14*(2), 311-324. doi:10.5194/bg-2016-254, 2016

D’ambrosio, L., Ziervogel, K., MacGregor, B., Teske, A., & Arnosti, C. (2014). Composition and enzymatic function of particle-associated and free-living bacteria: a coastal/offshore comparison. *The ISME journal*, *8*(11), 2167-2179. doi:10.1038/ismej.2014.67.

Dang, H., & Lovell, C. R. (2002). Numerical dominance and phylotype diversity of marine Rhodobacter species during early colonization of submerged surfaces in coastal marine waters as determined by 16S ribosomal DNA sequence analysis and fluorescence in situ hybridization. *Applied and environmental microbiology*, *68*(2), 496-504. doi:10.1128/AEM.68.2.496-504.2002.

De Jode, A., David, R., Haguenauer, A., Cahill, A. E., Erga, Z., Guillemain, D., ... & Chenuil, A. (2019). From seascape ecology to population genomics and back. Spatial and ecological differentiation among cryptic species of the red algae *Lithophyllum stictiforme/L. cabiochiae*, main bioconstructors of coralligenous habitats. *Molecular Phylogenetics and Evolution*, *137*, 104-113. [doi:10.1016/j.ympev.2019.04.005](https://doi.org/10.1016/j.ympev.2019.04.005)

Dickson, A. G., Sabine, C. L., & Christian, J. R. (2007). SOP 3b: Determination of total alkalinity in seawater using an open-cell titration. *Guide to best practices for ocean CO2 measurements*, *3*, 1-15.

Dishaw, L. J., Flores-Torres, J., Lax, S., Gemayel, K., Leigh, B., Melillo, D., ... & Gilbert, J. A. (2014). The gut of geographically disparate *Ciona intestinalis* harbors a core microbiota. *PLoS One*, *9*(4), e93386. doi:10.1371/journal.pone.0093386.

Dittami, S. M., Barbeyron, T., Boyen, C., Cambefort, J., Collet, G., Delage, L., ... & Tonon, T. (2014). Genome and metabolic network of “Candidatus Phaeomarinobacter ectocarpi” Ec32, a new candidate genus of Alphaproteobacteria frequently associated with brown algae. *Frontiers in genetics*, *5*, 241. doi:10.3389/fgene.2014.00241.

Dittmar, T., Koch, B., Hertkorn, N., & Kattner, G. (2008). A simple and efficient method for the solid‐phase extraction of dissolved organic matter (SPE‐DOM) from seawater. *Limnology and Oceanography: Methods*, *6*(6), 230-235. doi.org/10.4319/lom.2008.6.230

D'Onofrio, A., Crawford, J. M., Stewart, E. J., Witt, K., Gavrish, E., Epstein, S., ... & Lewis, K. (2010). Siderophores from neighboring organisms promote the growth of uncultured bacteria. *Chemistry & biology*, *17*(3), 254-264. doi: 10.1016/j.chembiol.2010.02.010.

Erwin, P. M., Pita, L., López-Legentil, S., & Turon, X. (2012). Stability of sponge-associated bacteria over large seasonal shifts in temperature and irradiance. *Applied and Environmental Microbiology*, *78*(20), 7358-7368. doi: 10.1128/AEM.02035-12.

Esteves, A. I., Amer, N., Nguyen, M., & Thomas, T. (2016). Sample Processing Impacts the Viability and Cultivability of the Sponge Microbiome. *Frontiers in Microbiology,* *12,* 7-499. doi: 10.3389/fmicb.2016.00499.

Flemer, B., Kennedy, J., Margassery, L. M., Morrissey, J. P., O’Gara, F., & Dobson, A. D. W. (2012). Diversity and antimicrobial activities of microbes from two Irish marine sponges, *Suberites carnosus* and *Leucosolenia* sp. *Journal of applied microbiology*, *112*(2), 289-301. doi: 10.1111/j.1365-2672.2011.05211.x.

Galli, G., Solidoro, C., & Lovato, T. (2017). Marine heat waves hazard 3D maps and the risk for low motility organisms in a warming Mediterranean Sea. *Frontiers in Marine Science*, *4*, 136. [doi.org/10.3389/fmars.2017.00136](https://doi.org/10.3389/fmars.2017.00136)

Gattuso, J., Epitalon, J., Lavigne, H., & Orr, J. (2024). seacarb: Seawater Carbonate Chemistry. R package version 3.3.3.,<https://github.com/jpgattuso/seacarb-git>.

Giovannelli, D., d'Errico, G., Manini, E., Yakimov, M., & Vetriani, C. (2013). Diversity and phylogenetic analyses of bacteria from a shallow-water hydrothermal vent in Milos island (Greece). *Frontiers in microbiology*, *4*, 184. doi: 10.3389/fmicb.2013.00184.

Goldsmith, D. B., Pratte, Z. A., Kellogg, C. A., Snader, S. E., & Sharp, K. H. (2019). Stability of temperate coral *Astrangia poculata* microbiome is reflected across different sequencing methodologies. *AIMS microbiology*, *5*(1), 62. doi:10.3934/microbiol.2019.1.62

Heijs, S. K., Sinninghe Damsté, J. S., & Forney, L. J. (2005). Characterization of a deep-sea microbial mat from an active cold seep at the Milano mud volcano in the Eastern Mediterranean Sea. *FEMS microbiology ecology*, *54*(1), 47-56. doi: 10.1016/j.femsec.2005.02.007.

Isaacs, L. T., Kan, J., Nguyen, L., Videau, P., Anderson, M. A., Wright, T. L., & Hill, R. T. (2009). Comparison of the bacterial communities of wild and captive sponge *Clathria prolifera* from the Chesapeake Bay. *Marine Biotechnology*, *11*, 758-770. doi: 10.1007/s10126-009-9192-3.

Jensen, S. I., Kühl, M., & Prieme, A. (2007). Different bacterial communities associated with the roots and bulk sediment of the seagrass *Zostera marina*. *FEMS microbiology ecology*, *62*(1), 108-117. doi:10.1111/j.1574-6941.2007.00373.x.

Kang, H. S., & Lee, S. D. (2009) *Ponticaulis koreensi*s gen. nov., sp. nov., a new member of the family Hyphomonadaceae isolated from seawater. *International journal of systematic and evolutionary microbiology*, *59*(12), 2951-2955. doi: 10.1099/ijs.0.011114-0.

Keller-Costa, T., Eriksson, D., Goncalves, J. M., Gomes, N. C., Lago-Leston, A., & Costa, R. (2017). The gorgonian coral *Eunicella labiata* hosts a distinct prokaryotic consortium amenable to cultivation. *FEMS Microbiology Ecology*, *93*(12), fix143. doi:10.1093/femsec/fix143.

Kirk Harris, J., Gregory Caporaso, J., Walker, J. J., Spear, J. R., Gold, N. J., Robertson, C. E., ... & Pace, N. R. (2013). Phylogenetic stratigraphy in the Guerrero Negro hypersaline microbial mat. *The ISME journal*, *7*(1), 50-60. doi:10.1038/ismej.2012.79.

Klindworth, A., Pruesse, E., Schweer, T., Peplies, J., Quast, C., Horn, M., & Glöckner, F. O. (2013). Evaluation of general 16S ribosomal RNA gene PCR primers for classical and next-generation sequencing-based diversity studies. *Nucleic acids research*, *41*(1), e1-e1. doi. org/ 10.1093/ nar/ gks808.

Kriwy, P., & Uthicke, S. (2011). Microbial diversity in marine biofilms along a water quality gradient on the Great Barrier Reef. *Systematic and Applied Microbiology*, *34*(2), 116-126. doi:10.1016/j.syapm.2011.01.003.

Kwiatkowski, L., Torres, O., Bopp, L., Aumont, O., Chamberlain, M., Christian, J. R., ... & Ziehn, T. (2020). Twenty-first-century ocean warming, acidification, deoxygenation, and upper-ocean nutrient and primary production decline from CMIP6 model projections. *Biogeosciences*, *17*(13), 3439-3470. [doi.org/10.5194/bg-17-3439-2020](https://doi.org/10.5194/bg-17-3439-2020)

Lachnit, T., Meske, D., Wahl, M., Harder, T., & Schmitz, R. (2011). Epibacterial community patterns on marine macroalgae are host‐specific but temporally variable. *Environmental microbiology*, *13*(3), 655-665. doi:10.1111/j.1462-2920.2010.02371.x.

Li, H., Yu, Y., Luo, W., Zeng, Y., & Chen, B. (2009). Bacterial diversity in surface sediments from the Pacific Arctic Ocean. *Extremophiles*, *13*, 233-246. doi:10.1007/s00792-009-0225-7.

Manea, E., Bramanti, L., Pezzolesi, L., Falace, A., Kaleb, S., Bongiorni, L., & Galand, P. E. (2025). Inter-and intraspecific diversity in bacterial communities associated with two crustose coralline algae from the NW Mediterranean Sea. *CoralReefs*, 1-20. doi.org/10.1007/s00338-024-02605-6

Martiny, J. B., Eisen, J. A., Penn, K., Allison, S. D., & Horner-Devine, M. C. (2011). Drivers of bacterial β-diversity depend on spatial scale. *Proceedings of the National Academy of Sciences*, *108*(19), 7850-7854. doi: 10.1073/pnas.1016308108.

Meron, D., Atias, E., Iasur Kruh, L., Elifantz, H., Minz, D., Fine, M., & Banin, E. (2011). The impact of reduced pH on the microbial community of the coral *Acropora eurystoma*. *The ISME journal*, *5*(1), 51-60. doi:10.1038/ismej.2010.102.

Meron, D., Atias, E., Iasur Kruh, L., Elifantz, H., Minz, D., Fine, M., & Banin, E. (2011). The impact of reduced pH on the microbial community of the coral *Acropora eurystoma*. *The ISME journal*, *5*(1), 51-60. doi:10.1038/ismej.2010.102.

Meron, D., Rodolfo-Metalpa, R., Cunning, R., Baker, A. C., Fine, M., & Banin, E. (2012). Changes in coral microbial communities in response to a natural pH gradient. *The ISME journal*, *6*(9), 1775-1785. doi:10.1038/ismej.2012.19.

Mou, X., Hodson, R. E., & Moran, M. A. (2007). Bacterioplankton assemblages transforming dissolved organic compounds in coastal seawater. *Environmental microbiology*, *9*(8), 2025-2037. doi:10.1111/j.1462-2920.2007.01318.x.

Musat, N., Werner, U., Knittel, K., Kolb, S., Dodenhof, T., Van Beusekom, J. E., ... & Amann, R. (2006). Microbial community structure of sandy intertidal sediments in the North Sea, Sylt-Rømø Basin, Wadden Sea. *Systematic and applied microbiology*, *29*(4), 333-348. doi:10.1016/j.syapm.2005.12.006.

Na, H., Kim, O. S., Yoon, S. H., Kim, Y., & Chun, J. (2011). Comparative approach to capture bacterial diversity of coastal waters. *The Journal of Microbiology*, *49*, 729-740. doi:10.1007/s12275-011-1205-z.

Nedashkovskaya, O. I., Kim, S. G., Stenkova, A. M., Kukhlevskiy, A. D., Zhukova, N. V., & Mikhailov, V. V. (2018). *Aquimarina algiphila* sp. nov., a chitin degrading bacterium isolated from the red alga Tichocarpus crinitus. *International Journal of Systematic and Evolutionary Microbiology*, *68*(3), 892-898. doi:10.1099/ijsem.0.002606.

Nei, M., & Kumar, S. (2000). Molecular evolution and phylogenetics. *Oxford university press*.

Nshimyimana, J. P., Freedman, A. J. E., Shanahan, P., Chua, L. C., & Thompson, J. R. (2017). Variation of bacterial communities with water quality in an urban tropical catchment. *Environmental science & technology*, *51*(10), 5591-5601. doi: 10.1021/acs.est.6b04737

Peña, V., De Clerck, O., Afonso-Carrillo, J., Ballesteros, E., Bárbara, I., Barreiro, R., & Le Gall, L. (2015). An integrative systematic approach to species diversity and distribution in the genus Mesophyllum (Corallinales, Rhodophyta) in Atlantic and Mediterranean Europe. *European journal of phycology*, *50*(1), 20-36. [doi: 10.1080/09670262.2014.981294](https://doi.org/10.1080/09670262.2014.981294)

Pezzolesi, L., Falace, A., Kaleb, S., Hernandez‐Kantun, J. J., Cerrano, C., & Rindi, F. (2017). Genetic and morphological variation in an ecosystem engineer, *Lithophyllum byssoides* (Corallinales, Rhodophyta). *Journal of Phycology*, *53*(1), 146-160. doi.org/ 10. 1111/ jpy.12837

Pezzolesi, L., Peña, V., Le Gall, L., Gabrielson, P. W., Kaleb, S., Hughey, J. R., ... & Rindi, F. (2019). Mediterranean *Lithophyllum stictiforme* (Corallinales, Rhodophyta) is a genetically diverse species complex: implications for species circumscription, biogeography and conservation of coralligenous habitats. *Journal of Phycology*, *55*(2), 473-492. doi.org/10. 1111/ jpy. 12837

Pratheepa, V., Alex, A., Silva, M., & Vasconcelos, V. (2016). Bacterial diversity and tetrodotoxin analysis in the viscera of the gastropods from Portuguese coast. *Toxicon*, *119*, 186-193. [doi.org/10.1016/j.toxicon.2016.06.003](https://doi.org/10.1016/j.toxicon.2016.06.003)

Rainey, F. A., Silva, J., Nobre, M. F., Silva, M. T., & da Costa, M. S. (2003). *Porphyrobacter cryptus* sp. nov., a novel slightly thermophilic, aerobic, bacteriochlorophyll a-containing species. *International journal of systematic and evolutionary microbiology*, *53*(1), 35-41. doi:10.1099/ijs.0.02308-0 Webster

Reale, M., Cossarini, G., Lazzari, P., Lovato, T., Bolzon, G., Masina, S., ... & Salon, S. (2022). Acidification, deoxygenation, and nutrient and biomass declines in a warming Mediterranean Sea. *Biogeosciences*, *19*(17), 4035-4065. doi.org/10.5194/bg-19-4035-2022

Rungrassamee, W., Klanchui, A., Maibunkaew, S., & Karoonuthaisiri, N. (2016). Bacterial dynamics in intestines of the black tiger shrimp and the Pacific white shrimp during *Vibrio harveyi* exposure. *Journal of invertebrate pathology*, *133*, 12-19. doi:10.1016/j.jip.2015.11.004.

Saitou, N., & Nei, M. (1987). The neighbor-joining method: a new method for reconstructing phylogenetic trees. *Molecular biology and evolution*, *4*(4), 406-425.

Schä, H., Bernard, L., Courties, C., Lebaron, P., Servais, P., Pukall, R., ... & Muyzer, G. (2001). Microbial community dynamics in Mediterranean nutrient-enriched seawater mesocosms: changes in the genetic diversity of bacterial populations. *FEMS microbiology ecology*, *34*(3), 243-253. doi:10.1111/j.1574-6941.2001.tb00775.x.

Sekar, R., Kaczmarsky, L. T., & Richardson, L. L. (2008). Microbial community composition of black band disease on the coral host *Siderastrea siderea* from three regions of the wider Caribbean. *Marine Ecology Progress Series*, *362*, 85-98. doi: 10.3354/meps07496

Shimane, Y., Tsuruwaka, Y., Miyazaki, M., Mori, K., Minegishi, H., Echigo, A., ... & Hatada, Y. (2013). *Salinisphaera japonica* sp. nov., a moderately halophilic bacterium isolated from the surface of a deep-sea fish, *Malacocottus gibber*, and emended description of the genus Salinisphaera. *International Journal of Systematic and Evolutionary Microbiology*, *63*(6), 2180-2185. doi:10.1099/ijs.0.047845-0.

Siboni, N., Martinez, S., Abelson, A., Sivan, A., & Kushmaro, A. (2009). Conditioning film and initial biofilm formation on electrochemical CaCO3 deposition on a metallic net in the marine environment. *Biofouling*, *25*(7), 675-683.

Singh, S. K., Kotakonda, A., Kapardar, R. K., Kankipati, H. K., Sreenivasa Rao, P., Sankaranarayanan, P. M., ... & Shivaji, S. (2015). Response of bacterioplankton to iron fertilization of the Southern Ocean, Antarctica. *Frontiers in Microbiology*, *6*, 863.

Sipkema, D., & Blanch, H. W. (2010). Spatial distribution of bacteria associated with the marine sponge *Tethya californiana*. *Marine Biology*, *157*, 627-638. doi: 10.1007/s00227-009-1347-2.

Sipkema, D., Holmes, B., Nichols, S. A., & Blanch, H. W. (2009). Biological characterisation of *Haliclona (? gellius*) sp.: sponge and associated microorganisms. *Microbial Ecology*, *58*, 903-920. doi:10.1007/s00248-009-9534-8.

Sipkema, D., Schippers, K., Maalcke, W. J., Yang, Y., Salim, S., & Blanch, H. W. (2011). Multiple approaches to enhance the cultivability of bacteria associated with the marine sponge *Haliclona (gellius)* sp. *Applied and Environmental Microbiology*, *77*(6), 2130-2140. doi:10.1128/AEM.01203-10.

Sunagawa, S., DeSantis, T. Z., Piceno, Y. M., Brodie, E. L., DeSalvo, M. K., Voolstra, C. R., ... & Medina, M. (2009). Bacterial diversity and White Plague Disease-associated community changes in the Caribbean coral *Montastraea faveolata*. *The ISME journal*, *3*(5), 512-521. doi:10.1038/ismej.2008.131.

Sunagawa, S., Woodley, C. M., & Medina, M. (2010). Threatened corals provide underexplored microbial habitats. *PloS one*, *5*(3), e9554. doi: 10.1371/journal.pone.0009554.

Tamura, K., Stecher, G., & Kumar, S. (2021). MEGA11: molecular evolutionary genetics analysis version 11. *Molecular biology and evolution*, *38*(7), 3022-3027. doi:10.1093/molbev/msab120

Teeling, H., Fuchs, B. M., Becher, D., Klockow, C., Gardebrecht, A., Bennke, C. M., ... & Amann, R. (2012). Substrate-controlled succession of marine bacterioplankton populations induced by a phytoplankton bloom. *Science*, *336*(6081), 608-611. doi:10.1126/science.1218344.

Tian, F., Yu, Y., Chen, B., Li, H., Yao, Y. F., & Guo, X. K. (2009). Bacterial, archaeal and eukaryotic diversity in Arctic sediment as revealed by 16S rRNA and 18S rRNA gene clone libraries analysis. *Polar Biology*, *32*, 93-103. doi:10.1007/s00300-008-0509-x

Tignat-Perrier, R., Bramanti, L., Giordano, B., van de Water, J. A. J. M., Manea, E., Allemand, D., Ferrier-Pagès, C. (2025). Microbiome dynamics in early life stages of the precious Mediterranean red coral Corallium rubrum. Environmental Microbiology Reports, 17(3), e70127. doi.org/10.1111/1758-2229.70127

Toupoint, N., Mohit, V., Linossier, I., Bourgougnon, N., Myrand, B., Olivier, F., ... & Tremblay, R. (2012). Effect of biofilm age on settlement of *Mytilus edulis*. *Biofouling*, *28*(9), 985-1001. doi:10.1080/08927014.2012.725202.

Vogt, J. C., Abed, R. M., Albach, D. C., & Palinska, K. A. (2018). Bacterial and archaeal diversity in hypersaline cyanobacterial mats along a transect in the intertidal flats of the Sultanate of Oman. *Microbial ecology*, *75*, 331-347. doi: 10.1007/s00248-017-1040-9.

Walmsley, T. A., Matcher, G. F., Zhang, F., Hill, R. T., Davies-Coleman, M. T., & Dorrington, R. A. (2012). Diversity of bacterial communities associated with the Indian Ocean sponge *Tsitsikamma favus* that contains the bioactive pyrroloiminoquinones, tsitsikammamine A and B. *Marine Biotechnology*, *14*, 681-691. doi: 10.1007/s10126-012-9430-y.ù

Webster, N. S., Soo, R., Cobb, R., & Negri, A. P. (2011). Elevated seawater temperature causes a microbial shift on crustose coralline algae with implications for the recruitment of coral larvae. *The ISME journal*, *5*(4), 759-770. doi:10.1038/ismej.2010.152.

Webster, N. S., Uthicke, S., Botté, E. S., Flores, F., & Negri, A. P. (2013). Ocean acidification reduces induction of coral settlement by crustose coralline algae. *Global change biology*, *19*(1), 303-315. doi:10.1111/gcb.12008.

Witt, V., Wild, C., & Uthicke, S. (2011). Effect of substrate type on bacterial community composition in biofilms from the Great Barrier Reef. *FEMS Microbiology Letters*, *323*(2), 188-195. doi:10.1111/j.1574-6968.2011.02374.x.

Witt, V., Wild, C., & Uthicke, S. (2012). Terrestrial runoff controls the bacterial community composition of biofilms along a water quality gradient in the Great Barrier Reef. *Applied and Environmental Microbiology*, *78*(21), 7786-7791. doi: 10.1128/AEM.01623-12.

Xu, R., Yang, Z. H., Wang, Q. P., Bai, Y., Liu, J. B., Zheng, Y., ... & Fan, C. Z. (2018). Rapid startup of thermophilic anaerobic digester to remove tetracycline and sulfonamides resistance genes from sewage sludge. *Science of the Total Environment*, *612*, 788-798. [doi.org/10.1016/j.scitotenv.2017.08.295](https://doi.org/10.1016/j.scitotenv.2017.08.295)

Yang, Z., & Li, Z. (2012). Spatial distribution of prokaryotic symbionts and ammoxidation, denitrifier bacteria in marine sponge *Astrosclera willeyana*. *Scientific Reports*, *2*(1), 528. doi: 10.1038/srep00528.

Yoshida-Takashima, Y., Nunoura, T., Kazama, H., Noguchi, T., Inoue, K., Akashi, H., ... & Takai, K. (2012). Spatial distribution of viruses associated with planktonic and attached microbial communities in hydrothermal environments. *Applied and environmental microbiology*, *78*(5), 1311-1320. doi:10.1128/AEM.06491-11.

Zhang, Y., Jiao, N., Sun, Z., Hu, A., & Zheng, Q. (2011). Phylogenetic diversity of bacterial communities in South China Sea mesoscale cyclonic eddy perturbations. *Research in Microbiology*, *162*(3), 320-329.doi:10.1016/j.resmic.2010.12.006.
